# ANGPTL4 stabilizes atherosclerotic plaques and modulates the phenotypic transition of vascular smooth muscle cells through KLF4 downregulation

**DOI:** 10.1038/s12276-023-00937-x

**Published:** 2023-02-13

**Authors:** Dong Im Cho, Min Joo Ahn, Hyang Hee Cho, Meeyoung Cho, Ju Hee Jun, Bo Gyeong Kang, Soo Yeon Lim, Soo Ji Yoo, Mi Ra Kim, Hyung-Seok Kim, Su-Jin Lee, Le Thanh Dat, Changho Lee, Yong Sook Kim, Youngkeun Ahn

**Affiliations:** 1 Cell Regeneration Research Center, Chonnam National University Hospital, Gwangju, South Korea; 2 Department of Cardiology, Chonnam National University Medical School, Gwangju, Republic of Korea; 3 Department of Cardiology, Chonnam National University Hospital, Gwangju, South Korea; 4 Department of Forensic Medicine, Chonnam National University Medical School, Gwangju, South Korea; 5 Department of Artificial Intelligence Convergence, Chonnam National University, Gwangju, South Korea; 6 Department of Nuclear Medicine, Chonnam National University Medical School & Hwasun Hospital, Gwangju, South Korea; 7 Biomedical Research Institute, Chonnam National University Hospital, Gwangju, South Korea

**Keywords:** Inflammation, Atherosclerosis, Atherosclerosis

## Abstract

Atherosclerosis, the leading cause of death, is a vascular disease of chronic inflammation. We recently showed that angiopoietin-like 4 (ANGPTL4) promotes cardiac repair by suppressing pathological inflammation. Given the fundamental contribution of inflammation to atherosclerosis, we assessed the role of ANGPTL4 in the development of atherosclerosis and determined whether ANGPTL4 regulates atherosclerotic plaque stability. We injected ANGPTL4 protein twice a week into atherosclerotic *Apoe−/−* mice and analyzed the atherosclerotic lesion size, inflammation, and plaque stability. In atherosclerotic mice, ANGPTL4 reduced atherosclerotic plaque size and vascular inflammation. In the atherosclerotic lesions and fibrous caps, the number of α-SMA(+), SM22α(+), and SM-MHC(+) cells was higher, while the number of CD68(+) and Mac2(+) cells was lower in the ANGPTL4 group. Most importantly, the fibrous cap was significantly thicker in the ANGPTL4 group than in the control group. Smooth muscle cells (SMCs) isolated from atherosclerotic aortas showed significantly increased expression of CD68 and Krüppel-like factor 4 (KLF4), a modulator of the vascular SMC phenotype, along with downregulation of α-SMA, and these changes were attenuated by ANGPTL4 treatment. Furthermore, ANGPTL4 reduced TNFα-induced NADPH oxidase 1 (NOX1), a major source of reactive oxygen species, resulting in the attenuation of KLF4-mediated SMC phenotypic changes. We showed that acute myocardial infarction (AMI) patients with higher levels of ANGPTL4 had fewer vascular events than AMI patients with lower levels of ANGPTL4 (*p* < 0.05). Our results reveal that ANGPTL4 treatment inhibits atherogenesis and suggest that targeting vascular stability and inflammation may serve as a novel therapeutic strategy to prevent and treat atherosclerosis. Even more importantly, ANGPTL4 treatment inhibited the phenotypic changes of SMCs into macrophage-like cells by downregulating NOX1 activation of KLF4, leading to the formation of more stable plaques.

## Cardiovascular disease: Stopping and stabilizing atherosclerotic plaques

Treatment with a protein that stabilizes existing plaques within blood vessels could help reduce the risk of future cardiovascular events in patients with atherosclerosis. These plaques arise in part from a change in the behavior of the muscle cells within the walls of the blood vessels, which leads to the accumulation of lipids and other biomolecules and creates conditions that can ultimately result in a heart attack or stroke. Researchers led by Youngkeun Ahn and Yong Sook Kim at Chonnam National University Hospital, Gwangju, South Korea, have shown that they can counter this process in a mouse model of atherosclerosis by treatment with a protein called ANGPTL4. This molecule keeps vascular muscle cells in a state that prevents further plaque formation, while stabilizing existing plaques and countering the inflammatory processes that can further accelerate the cardiovascular disease.

## Introduction

Atherosclerosis, a complex vascular disorder, develops in the context of dyslipidemia and chronic inflammation and is the main contributor to cardiovascular mortality. In response to injury or inflammatory stimuli, circulating monocytes are recruited to activated endothelial cells by adhesion molecules such as monocyte chemoattractant protein-1 (MCP-1) and vascular cell adhesion molecule-1 (VCAM-1). These monocytes differentiate into macrophages to take up excessive lipids through scavenger receptors and form fatty streaks. In the lesion, activated vascular smooth muscle cells (VSMCs) become highly proliferative and migrate to the subendothelial space to form the tunica intima, and the phenotype of the VSMCs is shifted to the synthetic type from the contractile type to contribute to atheroma formation. This dynamic interplay between endothelial cells, VSMCs, and macrophages is altered in vascular pathologies such as atherosclerosis.

Thin-cap fibroatheroma, which has an increased risk of thrombosis, is a kind of vulnerable plaque^1,2^. Vulnerable plaques are at high risk of cardiovascular events such as acute myocardial infarction (AMI) and stroke^3^. Despite recent advances in the study of plaque biology, the mechanisms and contributing factors controlling the stability of atherosclerotic lesions remain unclear.

Clinical interventions for atherosclerosis include mostly lipid-lowering therapies, of which statins are the most widely used. Statins decrease C-reactive protein (CRP) concentrations, increase the collagen content of atherosclerotic plaques, change endothelial function, and reduce monocyte recruitment and macrophage accumulation in plaques^4–6^.

The results of many clinical trials attest to the diverse roles of statins^7^. Moderate or intensive statin therapy decreases low-density lipoprotein (LDL) cholesterol^8^, increases atheroma stabilization^9^, and enhances the regression of coronary plaque volume^10^. The results of the Study of Coronary Atheroma by Intravascular Ultrasound (SATURN) trial suggest that plaque regression occurs in more than 60% of patients treated with rosuvastatin or atorvastatin^11^. Unfortunately, atherosclerosis continues to progress in up to one-third of patients despite intensive statin therapy^12^.

In 2017, the Canakinumab Anti-inflammatory Thrombosis Outcomes Study (CANTOS) showed that inhibition of inflammatory interleukin-1β (IL-1β) can significantly reduce the number of cardiovascular events independently of lipid-lowering, but the mechanisms underlying this anti-inflammatory therapy remain unclear^13^. The Cardiovascular Inflammation Reduction Trial (CIRT) showed that treatment with low-dose methotrexate fails to lower cardiovascular event rates^14^. Then, in 2019, The Colchicine Cardiovascular Outcomes Trial (COLCOT) suggested that treatment with colchicine reduces the incidence of adverse coronary and cerebral atherothrombotic events^15^.

Since we have shown the cardioprotective effects of anti-inflammatory angiopoietin-like 4 (ANGPTL4) in a mouse model of MI^16^, we were interested in whether ANGPTL4 could exert a protective function during the development of atherosclerosis. ANGPTL proteins are structurally similar to angiopoietins that play a role in a wide array of biological functions, including the regulation of lipid and glucose metabolism, hematopoietic stem cell expansion, chronic inflammation, angiogenesis, and wound healing. Therefore, to investigate the effect of ANGPTL4 on inflammatory vascular disease, we performed a comprehensive evaluation of atherogenesis, including inflammatory phenotype and plaque stability, in an *Apoe*-/- mouse model of high-fat diet-induced atherosclerosis. Furthermore, to understand the clinical implications of our studies, we measured circulating levels of ANGPTL4 in patients with cardiovascular disease to determine the association of ANGPTL4 with clinical outcomes.

Recent data indicate that VSMCs in atherosclerotic lesions undergo a phenotypic transition to macrophage-like cells that express both macrophage and SMC markers and promote inflammation and enhanced atherogenesis^17^. In addition, activation of the transcription factor KLF4 by oxidized phospholipids promotes phenotypic modulation of VSMCs in mouse and human atherosclerotic plaques^18^. Other functions of VSMCs include sustaining vascular wall reactive oxygen species (ROS) generation, inflammation, and matrix remodeling. Thus, VSMCs are believed to play a major role in atherogenesis and the evolution of atherosclerotic lesions^19^.

In this study, we demonstrated that ANGPTL4 administration significantly reduced atherosclerotic lesion size, macrophage content, vascular inflammation, and phenotypic transition of smooth muscle cells, contributing to plaque stabilization by downregulating NADPH oxidase 1 (NOX1) activation of KLF4 in an atherosclerosis mouse model.

## Materials and methods

### Human study population

Study subjects provided written informed consent before enrollment. For immunohistochemical studies, human left anterior descending artery (LAD) specimens were obtained from the Department of Forensic Medicine, Chonnam National University Medical School, after approval of the research protocol used in this study. The Institutional Review Board of Chonnam National University Medical School and Hospital waived the requirement to obtain informed consent for the use of the forensic samples because of the inaccessibility to personally identifiable information and the fact that there were no tests for heritable traits in accordance with Article 33 of the Enforcement Regulations provided by the Korean government.

A total of 253 AMI patients who were admitted to Chonnam National University Hospital between January 2016 and July 2019 and who consented to the use of their blood samples for scientific purposes were screened. Forty-four patients were excluded due to a previous history of AMI or angina treated with percutaneous coronary intervention (PCI). Two more patients who died during the index hospitalization were also excluded. All patients had a successful PCI. Blood samples were obtained from all patients during PCI, and plasma ANGPTL4 levels were measured using an enzyme-linked immunosorbent assay (ELISA) kit (EHANGPTL4, Thermo Fisher Scientific). Patients were divided into two groups with high or low ANGPTL4 according to the median ANGPTL4 level. We compared clinical outcomes between the two groups during the follow-up period. The median duration of follow-up was 1.9 years (interquartile range, 1.3–3.3 years). This study was a single-center study, and the study protocol was approved by the Chonnam National University Hospital Institutional Review Board (BTMP-2020-330).

### Study definitions and endpoints

AMI was defined as the presence of acute myocardial injury detected by abnormal levels of cardiac biomarkers and angiographically proven atherothrombotic coronary artery disease (CAD). Cardiology specialists collected the patients’ medication lists and medical histories, such as the presence of hypertension, diabetes mellitus, and dyslipidemia. All laboratory variables were measured upon admission, except for lipid profiles, which were obtained after at least 9 h of fasting within 24 h of hospitalization. The baseline left ventricular ejection fraction (LVEF) was determined by two-dimensional echocardiography performed before or immediately after PCI. Coronary blood flow before and after PCI was classified by the thrombolysis in myocardial infarction (TIMI) score, and coronary lesion complexity was based on the American College of Cardiology (ACC)/American Heart Association (AHA) definitions. Patients who underwent PCI received 300 mg of aspirin and 600 mg of clopidogrel, 60 mg of prasugrel, or 180 mg of ticagrelor as a loading dose before PCI. Doses of 50 to 70 U/kg of unfractionated heparin were used before or during PCI to maintain an activated clotting time of 250 to 300 s. After PCI, 100 to 300 mg of aspirin and 75 mg of clopidogrel, 5 to 10 mg of prasugrel, or 45 to 90 mg of ticagrelor were prescribed daily. All patients had coronary lesions with at least 50% stenosis by quantitative coronary analysis. The study endpoint was vascular events associated with plaque instability, which consisted of recurrent AMI, stent thrombosis, and ischemic cerebral infarction. Recurrent MI was defined as the development of recurrent angina symptoms with new 12-lead electrocardiographic changes or increased cardiac-specific biomarkers.

### Mouse lines and atherosclerosis models

C57Bl/6 *Apoe−/−* mice were purchased from Jackson Laboratories (Bar Harbor, ME). Male mice were used for experiments beginning at 8 to 10 weeks of age. Accelerated atherosclerosis was induced by feeding the mice for 8 weeks with a Paigen diet containing 16% fat, 1.25% cholesterol, and 0.5% cholate (D12336, Research Diets) and with a Western diet containing 0.15% cholesterol (D12079B, Research Diets). *Apoe*−/− mice receiving a high-fat diet were intraperitoneally injected with 2 μg of ANGPTL4 (4880-AN, R&D Systems) two times per week for 8 weeks. The mice were bred and maintained under pathogen-free conditions at Chonnam National University Medical School animal facilities. All experiments were performed after approval by our local ethics committee at Chonnam National University Medical School (CNU IACUC-H-2018-55). *Ldlr−/−* mice were provided by the National Institute of Food and Drug Safety Evaluation (Chungcheongbukdo, Korea). The mouse genotyping primers are described in Supplementary Table 2.

### Cell culture

Human aortic SMCs (CC-2571, Lonza) were used between passages 4 and 10. Human aortic SMCs were maintained in SmGM 2 Smooth Muscle Cell Growth Medium 2 (CC-3182, Lonza). Human induced pluripotent stem cells (hiPSCs) were differentiated into hiPSC-derived cardiac progenitor cells (hiPSC-CPCs) using a STEMdiff™ Cardiomyocyte Differentiation kit (05010, STEMCELL). First, hiPSCs were incubated for 2 days in STEMdiff™ Cardiomyocyte Differentiation Medium A, for 2 days in STEMdiff™ Cardiomyocyte Differentiation Medium B, and for 4 days in STEMdiff™ Cardiomyocyte Differentiation Medium C. On Day 8, hiPSC-CPCs were cultured in advanced DMEM/F-12 medium (12634010, Gibco®, Life Technologies) with 5 μM CHIR99021 (S2924, Selleck Chemicals) and 2 μM retinoic acid (R2625, Sigma‒Aldrich) for 3 days and recovered in advanced DMEM/F-12 medium for 4 days. Human iPSC-epicardial cells (hiPSC-EPCs) were cultured in SMC growth medium with PDGF-BB (10 ng/ml, MBS142119, MyBioSource) and TGFβ1 (2 ng/ml, 7754-BH, R&D Systems) for 12 days. Human iPSC-derived smooth muscle cells (hiPSC-SMCs) (αSMA^+^/SM22α^+^) were identified by qPCR and immunofluorescence staining. For mouse bone marrow-derived macrophages (BMDMs), mononuclear cells were isolated from mouse bone marrow and cultured for 7 days in macrophage differentiation medium (supplemented with 30% L929 cell-conditioned medium, 20% fetal bovine serum [FBS; 16000044, Gibco], and 50% RPMI-1640 [11875-093, Gibco]). L929 cell-conditioned medium was prepared by growing L929 cells in RPMI-1640 medium containing 10% FBS for 10 days. The medium containing macrophage colony-stimulating factor secreted by the L929 cells was harvested and passed through a 0.22-mm filter. BMDMs were cultured in RPMI supplemented with 10% FBS for 3 days. Conditioned medium was collected and used immediately or stored at −80 °C.

### RNA isolation and real-time PCR

Tissue and cell RNAs were extracted using TRIzol (15596018, Life Technologies) and converted to cDNA using an Applied Biosystems High-Capacity cDNA reverse transcription kit (4368814, Life Technologies) according to the manufacturer’s instructions. Real-time PCR was performed using a QuantiTect SYBR Green PCR kit (204143, Qiagen) and Corbett Research Rotor-Gene RG-3000 Real-Time PCR System. The mouse primers are listed in Supplementary Table 1.

### Analysis of atherosclerotic lesions

For the analysis of mouse atherosclerotic lesions, aortas were harvested, cleaned of the adventitia, dissected longitudinally along the greater and lesser curvature for bilateral presentation, pinned, and en face-stained with Oil red O (O1391, Sigma‒Aldrich) for lipid measurement at the surface of the vascular wall. The images were captured using a digital camera (Samsung, Korea). The aortas and aortic roots were stained for lipid deposition with Oil red O. In brief, hearts with aortic roots were embedded in optimal cutting temperature (OCT) compound (3801480, Leica) for cryosectioning (Leica CM1850). Atherosclerotic lesions were quantified in 10-μm transverse sections, and the averages were calculated from 3 to 5 sections. The slides were stained using Oil red O for lipid deposition, hematoxylin-eosin (H&E, ab245880, Abcam) for aortic plaque necrosis, Masson’s trichrome staining (ab150686, Abcam) for the aortic fibrous cap, and picrosirius red staining (150681, Abcam) for analysis of collagen content. For histological analysis, the images were quantified as the average lesion area, which was measured using a color image analysis system (NIS-Elements Imaging Software, Nikon, Japan). For analysis of the cellular composition or inflammation of atherosclerotic lesions, sections were stained with an antibody against CD68 (ab125212, Abcam), Mac2 (CL8942AP, Cedarlane), α-SMA (A2547, Sigma), transgelin (SM22α) (Ab14106, Abcam), or SM-MHC (TA323338, OriGene). Foam cells were stained with 10 μg/ml BODIPY 493/503 (D3922, Invitrogen) at the same time as secondary antibody incubations. The nuclei were counterstained using 4’,6-diamidino-2-phenylindole (DAPI), and the positive areas were quantified with a color image analysis system.

### Isolation of murine aortic vascular smooth muscle cells

Aortic VSMCs were isolated by procedures described in Dr. Gary Owens’s Laboratory. Briefly, aortas from the root to the iliac bifurcation were dissected from adult *Apoe−/−* mice and opened longitudinally. The aortas were sequentially washed with 1% penicillin/streptomycin in HBSS (14025-092, Gibco), and then the endothelial cells and the adventitia were gently removed. Aortas were cut into small pieces and incubated at 37 °C in 5% CO_2_ in an enzyme solution (collagenase type II [LS004176, Worthington], elastase [E1250, Sigma], 1% penicillin/streptomycin, 20% FBS in DMEM/F-12 medium) for 1 h. Aortas were cultured in a medium with 20% FBS and 1% penicillin/streptomycin. After 7 days, the aortic medium of the SMCs was replaced with a fresh medium. Aortic SMCs were transferred to a fresh medium containing 10% FBS after passage 3 depending on their viability.

### Loading with water-soluble cholesterol

Aortic VSMCs were serum starved in DMEM with 0.2% BSA for 24 h and then loaded with cholesterol. SMCs were incubated with cholesterol-methyl-β-cyclodextrin complexes (Chol:MβCD, 10 μg/mL) (C4951, Sigma) and 0.2% BSA (A9418, Sigma) in DMEM for 3 days and then prepared for experiments.

### Flow cytometry

Freshly isolated aortas were resuspended in FACS buffer (PBS containing 1% FBS and 2 mM EDTA) and stained with conjugated antibody for 20 to 30 min at 4 °C. Cells were washed and resuspended in FACS buffer for flow cytometric analyses in which inflammatory cell populations were designated, following gating/stratification of their marker profile. The aortas were cut into small pieces and digested in an enzyme mixture containing 450 U/mL collagenase I (C0130, Sigma‒Aldrich), 125 U/mL collagenase XI (C7657, Sigma‒Aldrich), 60 U DNase I (DN25, Sigma‒Aldrich), and 60 U/mL hyaluronidase (2592, Worthington Laboratories) in PBS with Ca^2+^/Mg^2+^ for 60 min at 37 °C with gentle shaking. After incubation, the digestion mixture was homogenized through a 70-μm nylon mesh. The digestion mixture was centrifuged at 2000 rpm for 15 min at 4 °C, and the cells were simultaneously stained with antibodies at 4 °C for 15 min and then washed and resuspended in a staining buffer. All antibodies are listed in Supplementary Table 3. The cell suspensions were analyzed with a BD FACSCANTO II flow cytometry system (BD Biosciences), and postacquisition analysis was performed with FlowJo7 software (Tree Star).

### Foam cell formation assay

BMDMs from *Apoe−/−* mice were fixed with 4% paraformaldehyde (DN-4310, DANA Korea) for 20 min and then stained with Oil red O for 20 min at 37 °C. The BMDMs were washed with PBS, rinsed in 60% isopropanol (109634, Merck Millipore) for 15 s, and observed using a fluorescence microscope (Nikon, Japan). The Oil red O content was quantified using a color image analysis system (NIS-Elements Imaging Software, Nikon).

### Oxidized LDL uptake assay

The assay was performed using an oxidized LDL (oxLDL) uptake assay kit (601180, Cayman) or DiI-oxLDL (L34358, Invitrogen). The BMDMs from *Apoe−/−* mice were incubated with oxLDL-DyLight 488 or Dil-oxLDL (10 μg/mL) for 4 h. After unbound LDL was washed out, the cells were fixed with 4% paraformaldehyde. The fluorescent cells were observed using an Eclipse Ti2 microscope (Nikon, Japan).

### Immunoblot analysis

Cells were washed with ice-cold phosphate-buffered saline and lysed in PRO-PREP Protein Extraction Solution (17081, iNtRON Biotechnology) on a rotation wheel for 20 min at 4 °C. After centrifugation at 10,000×*g* for 10 min, the supernatant was prepared as a protein extract. Equal amounts of proteins were fractionated by electrophoresis on 8 or 10% acrylamide gels and were transferred onto a polyvinylidene fluoride membrane (IPVH00010, Millipore), followed by blotting with primary antibody and horseradish peroxidase-conjugated secondary IgG antibodies. Protein expression was detected using an Image Reader (LAS-3000 Imaging System, Fuji Photo Film).

### Human aorta smooth muscle cell proliferation assay

To stain proliferating cells with Ki67, HAoSMCs were fixed in 4% paraformaldehyde for 15 min. Then, the cell membrane was penetrated by 0.25% Triton X-100 for 10 min and blocked with normal goat serum for 1 h, followed by Ki67 antibody (ab15580, Abcam) staining at 4 °C overnight. Subsequently, the cells were incubated with secondary antibodies conjugated with Alexa-488 (A11034, Molecular Probes) or Alexa-594 (A11037, Molecular Probes) for 1 h, followed by mounting with 4,6-diamidino-2-phenylindole (D1306, Molecular Probes). The stained cells were visualized using a fluorescence microscope. Additionally, HAoSMC proliferation was analyzed using a colorimetric bromodeoxyuridine (BrdU) ELISA kit (6813, Cell Signaling Technology) according to the manufacturer’s manual. Cells were seeded into 96-well plates at a density of 1 × 10^4^ cells/well. The cells were then pretreated with or without different concentrations of recombinant human ANGPTL4 (4487-AN, R&D Systems) without serum for 3 h and stimulated with platelet-derived growth factor-BB (PDGF-BB, 20 ng/mL, MBS142119, MyBioSource) for 24 h. The cells were then labeled with BrdU labeling reagent for 3 h. After fixation, the cells were incubated with an anti-BrdU antibody for 1 h. After washing, HRP-conjugated secondary antibody substrate TMB was added to each well, and the plates were incubated at room temperature for 30 min. The absorbance was measured at a dual wavelength of 450/550 nm.

### Blood lipid and cytokine analyses

Pooled plasma samples from mice were assayed for leptin (MOB00B, R&D systems), IL-6 (BMS603-2, Invitrogen) IL-1β (BMS6002, Invitrogen), IL-18 (BMS618-3, Invitrogen), and ANGPTL4 (EHANGPTL4, Invitrogen) using ELISA kits.

### Luciferase reporter assay

For the luciferase assay, transfections were performed with Lipofectamine 2000 transfection reagent (Invitrogen, 11668-019) in human aortic SMCs. The *Firefly* luciferase vector was cotransfected with the KLF4 promoter construct as a control for transfection efficiency. The cells were then pretreated with or without different concentrations of recombinant human ANGPTL4 (4487-AN, R&D Systems) and stimulated with oxLDL (10 μg/mL) (L34357, Invitrogen) or recombinant human tumor necrosis factor α (TNF-α; 10 ng/mL) (210-TA, R&D Systems) for 24 h. The promoter activity was measured using the Pierce Gaussia-Firefly Luciferase Dual Assay kit (16182, Thermo Fisher Scientific) and a Sirius Luminometer (Berthold Technologies) according to the manufacturer’s instructions.

### ROS detection

In cultured VSMCs treated with TNFα (100 ng/ml), ROS levels were determined immediately after sample collection. Cellular ROS levels were assessed by measuring CM-H_2_DCFDA (C-6827, Invitrogen) fluorescence. CM-H2DCFDA was added to the culture medium at a final concentration of 13 μM, and the cells were incubated for 10 min at 37 °C. Cells were then washed three times with prewarmed PBS, and fluorescence images were taken using an Eclipse Ti2 microscope (Nikon, Japan). Superoxide generation in aortic root sections from *Apoe−/−* mice was evaluated by measuring DHE (dihydroethidium, D11347, Invitrogen) fluorescence. The aortic root sections were incubated with 5 μM DHE for 10 min in the dark and observed using a fluorescence microscope (Nikon, Japan). Fluorescent and grayscale images were analyzed with NIH ImageJ 1.53 software to determine the mean fluorescence density.

### Scanning electron microscopy

Aortas isolated from *Apoe−/−* mice for scanning electron microscope analysis were fixed in situ with 3% glutaraldehyde (G5882, Sigma) and harvested. Fixed aortas were postfixed with 2% osmium tetroxide (OsO4, pH 7.4) (201030, Sigma) and dehydrated with graded ethanol (30–100%). Chemical dehydration was achieved by incubation of the samples with 50% hexamethyldisilazane (440191, Sigma) for 20 min and an additional 20 min with fresh 100% hexamethyldisilazane. Aortic samples were prepared using a standard procedure for scanning electron microscopy, and photographs were taken in a routine manner.

### High-resolution optical resolution photoacoustic microscopy (OR-PAM)

A nanosecond laser system (SPOT-10-200-532, Elforlight, Daventry, UK) was used at the primary wavelength of 532 nm with a duration of 6 ns. A single-mode optical fiber (P1-405BPM-FC-1, Thorlabs, NJ, USA) transferred the laser beam, which was collimated to 2 mm in diameter. The laser beam was focused by a doublet lens (AC254-060-A, Thorlabs, NJ, USA), reflected 45° by a custom aluminum-coated prism in the beam combiner. The focused laser beam conducted three-dimensional scanning with a MEMs scanner (OptichoMS-001, Opticho Inc., Ltd., Pohang, Korea) on the *x-axis* and two linear stages (L-509, Physik Instruments (PI), Karlsruhe, Germany) on the *x-y axis*. The laser beam was used to irradiate the sample, and an acoustic wave was generated and passed through the beam combiner. The acoustic signal was detected using a high-frequency transducer (V214-BC-RM, 50 MHz center frequency, Olympus) as the analog signal. Then, an RF amplifier (ZX60-3018G-S+, Mini-Circuit, Brooklyn, NY, USA) and a low-pass crystal filter (CLPFL-0050, 50 MHz, CRYSTEK, Fort Myers, Florida, USA) were used as preprocessing steps to clean the analog signal. A high-speed digitizer (ATS9371, AlazarTech, Pointe-Claire, QC, Canada) converted the analog signal to a digital signal. The OR-PAM data were recorded, showing the *x-y* location and depth information of the digital acoustic signal. The control and preprocessing steps were stepped by a LabVIEW program (National Instruments, Austin, TX, USA). The measured lateral resolution was 12 µm, and the axial resolution was 27 µm.

### Statistical analysis

For the human study, continuous variables are presented as the means ± standard deviations or medians and interquartile ranges and were compared using the unpaired *t*-test or the Mann–Whitney rank-sum test. Discrete variables are expressed as counts and percentages and were analyzed with Pearson’s chi-square test or Fisher’s exact test. Kaplan–Meier curves were constructed for comparison of the study endpoints between the two groups, and differences were assessed with the log-rank test. Cox proportional hazards regression was used to identify factors associated with an increased risk of vascular events. Factors associated with mortality with a *p* value of less than 0.20 in the univariate analysis were entered into the multivariate model, and nonsignificant factors were removed using a backward-selection procedure. All analyses were two-tailed, and all variables were considered significant at the level of *p* < 0.05. All statistical analyses were performed using SPSS for Windows ver. 26.0 (IBM SPSS Inc., Chicago, IL, USA).

Differences between the experimental and control groups in the animal study were analyzed by Student’s *t-*test or one-way ANOVA with Bonferroni’s multiple-comparisons test.

All statistical analyses were performed using SPSS for Windows ver. 26.0 (IBM SPSS Inc., Chicago, IL, USA). A *P* value less than 0.05 was considered significant.

## Results

### ANGPTL4 administration attenuates the progression of atherosclerotic plaques in atherosclerotic mice

**Fig. 1:**
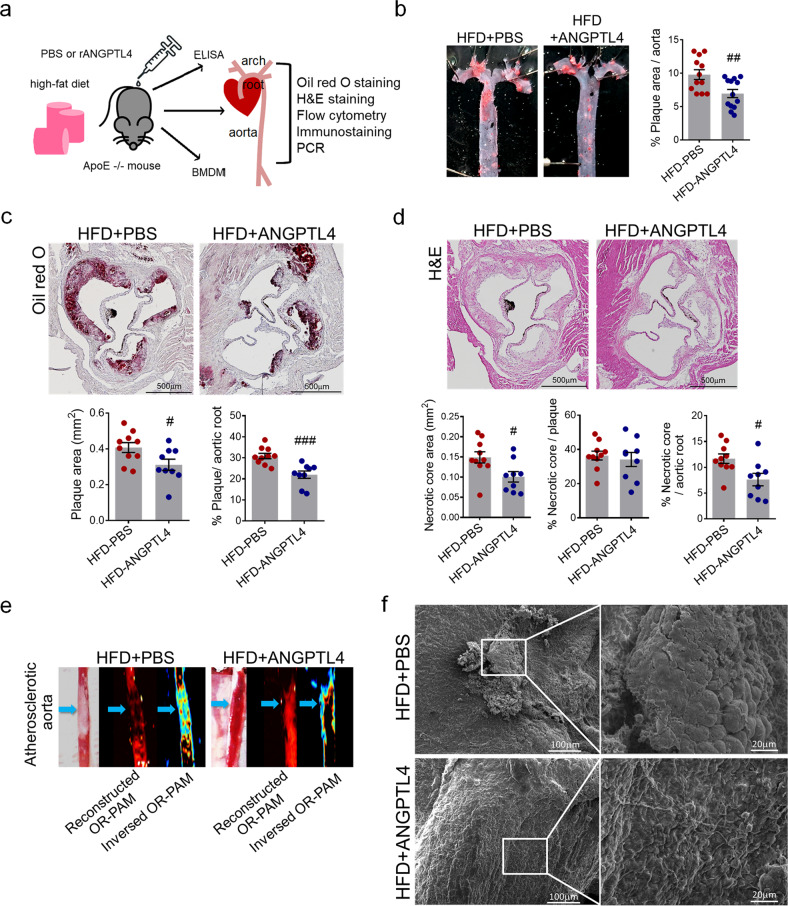
Effects of ANGPTL4 administration on atherosclerotic progression in the atherosclerosis *Apoe−/*− mouse model. **a** Experimental scheme. *Apoe−/−* mice were fed a high-fat diet (HFD) with an injection of PBS or recombinant ANGPTL4 protein (2 μg per mouse, intraperitoneally twice a week) for 8 weeks. Blood, BMDMs, aortic roots, and aortas were collected for further analyses. **b** Representative Oil red O-stained aortas from PBS-injected and ANGPTL4-injected *Apoe−/*− mice fed an HFD for 8 weeks. The relative atherosclerotic plaque area was quantified. **c**, Lesion area measured in Oil red O-stained cross sections of the aortic roots from the PBS and ANGPTL4 groups. The plaque area was measured and quantified as the relative size of the plaque to the aortic area. Scale bar, 500 μm. **d** Representative images are shown for H&E-stained aortic roots from the PBS and ANGPTL4 groups. The necrotic core area, qualified by the anucleated area, was measured and quantified as the relative size of the necrotic core to the plaque or aortic root. Scale bar, 500 μm. **e** Representative images of atherosclerotic aortas were obtained using high-resolution optical resolution photoacoustic microscopy (OR-PAM). Blue arrows indicate atherosclerotic plaques. **f** Scanning electron microscope images showing the aorta surface isolated from the PBS and ANGPTL4 groups. Boxed areas on the aortic surface depict atherosclerotic plaques at higher magnification. Scale bar, 20 μm. Data were presented as the mean ± SEM. #*p* < 0.05, ##*p* < 0.01, ###*p* < 0.001 (by Student’s *t*-test).

To determine the effect of ANGPTL4 administration on atherosclerotic lesion development, *Apoe*−/− mice fed a high-fat diet were injected with 2 μg of ANGPTL4 two times per week for 8 weeks and compared with mice injected with PBS alone. The experimental procedures are summarized in Fig. 1a. Atherosclerosis was induced by feeding a high-fat diet for 8 weeks. From the beginning of the high-fat diet, mice were intraperitoneally injected with PBS or ANGPTL4 protein twice per week. The entire aorta, aortic root, blood, BMDMs, and VSMCs were collected for further analyses.

*En face* analysis of aortic lesions in the entire aorta and quantification of atherosclerotic lesions showed that the relative plaque size was smaller in the ANGPTL4 group than in the PBS group (Fig. 1b). Oil red O-stained aortic roots showed that both the plaque area (0.408 ± 0.087 vs. 0.315 ± 0.095 mm^2^, *p* < 0.05) and relative plaque size (30.860 ± 4.031% vs. 21.989 ± 5.236%, *p* < 0.001) were smaller in the ANGPTL4 group (Fig. 1c). The necrotic core was measured through H&E staining, and the area of the necrotic core and percentage of the necrotic core of the aortic root were significantly smaller in the ANGPTL4 group than in the PBS group (11.680 ± 2.899% vs. 7.622 ± 3.604%, *p* < 0.05, Fig. 1d).

The atheroprotective effect of ANGPTL4 was additively validated in an *Ldlr*−/− mouse atherosclerosis model. In *Ldlr*−/− mice fed a high-fat diet for 16 weeks, atherosclerotic lesions and necrotic core areas were smaller in the ANGPTL4 group than in the PBS group (Supplementary Fig. 1a–c). In addition to aortic roots, aortas also showed smaller atherosclerotic lesions in the ANGPTL4 group than in the PBS group (Supplementary Fig. 1d).

The plaque within the aorta was visualized using an optical resolution photoacoustic microscopy system, which showed less plaque in the ANGPTL4 group than in the PBS group (Fig. 1e). Scanning electron microscopy was used to visualize the luminal surfaces of mouse aortas. After 8 weeks of the high-fat diet, endothelial deposits and atherosclerotic plaque on the aortic surface were decreased in the ANGPTL4 group (Fig. 1f). These results indicated that ANGPTL4 administration attenuates atherosclerotic plaque progression in *Apoe−/−* mice.

### ANGPTL4 administration relieves inflammatory and atherogenic phenotypes

**Fig. 2:**
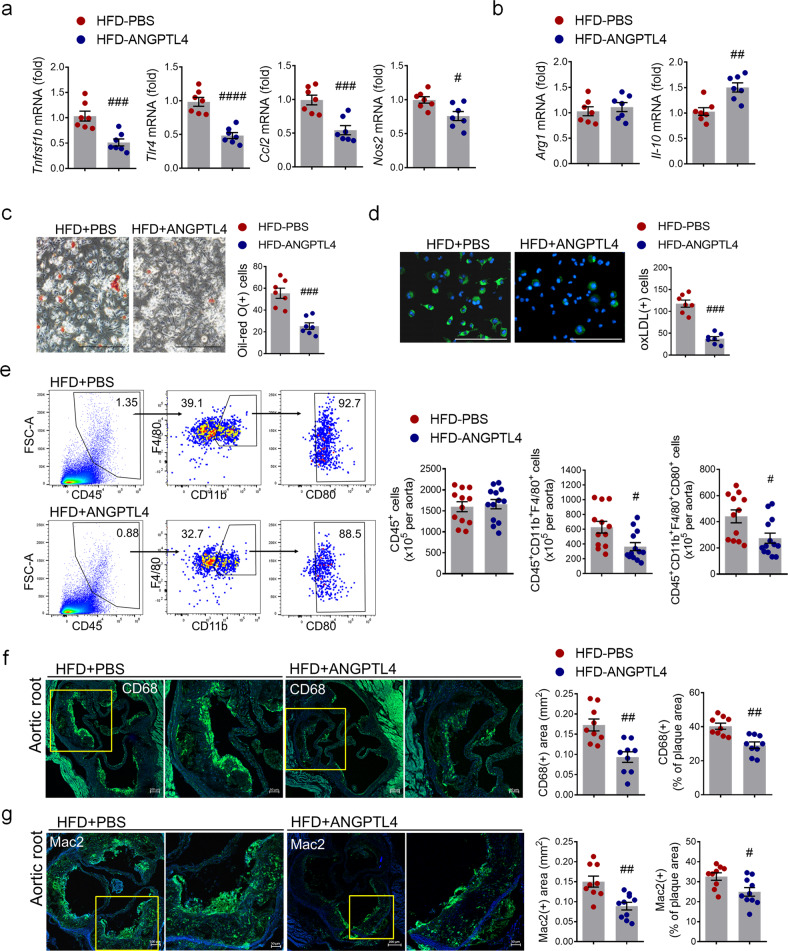
Effects of ANGPTL4 on macrophage function in atherosclerosis. **a**, **b** Proinflammatory and anti-inflammatory gene expression were analyzed by real-time PCR in BMDMs isolated from *Apoe−/−* mice treated with PBS or ANGPTL4. **c** BMDMs from the PBS and ANGPTL4 groups were analyzed using Oil red O staining. Scale bar, 200 μm. **d** BMDMs from the PBS and ANGPTL4 groups were treated with oxLDL-DyLight 488 for 24 h, and then oxidized LDL uptake was analyzed. Scale bar, 200 μm. **e** Flow cytometry analyses of single-cell aortic suspensions isolated from the PBS and ANGPTL4 groups. Inflammatory macrophages were quantified by the number of CD80^+^ cells among the CD45^+^F4/80^+^CD11b^+^ population. **f**, **g** The content of macrophages in aortic root sections from the two groups was determined by immunohistochemical staining with anti-CD68 antibody (**f**) and anti-Mac2 antibodies (**g**). Representative images are shown, and CD68-positive areas (**f**) and Mac2-positive areas (**g**) were measured and quantified as a percentage of the plaque area. Data were presented as the mean ± SEM. #*p* < 0.05, ##*p* < 0.01, ###*p* < 0.001, ####*p* < 0.0001 (by Student’s *t*-test).

Macrophages are an important component of atherosclerotic lesions and are associated with the progression of plaques. To determine whether ANGPTL4 contributes to macrophage function and gene expression in atherosclerosis, BMDMs from PBS- or ANGPTL4-injected atherosclerotic mice were isolated, and the mRNA expression of inflammation-related markers was compared. The expression levels of inflammatory markers such as Tnfrsf11b, Tlr4, Ccl2, and Nos2 were lower, whereas that of anti-inflammatory IL-10 was higher in BMDMs isolated from the ANGPTL4 group (Fig. 2a, b). Next, the atherogenic features of macrophages were examined using Oil red O staining and a fluorescence oxidized LDL uptake assay (Fig. 2c, d). Foam cell formation and oxidized LDL uptake were reduced in BMDMs of the ANGPTL4 group.

To assess the macrophage phenotype, we isolated single cells from the entire aorta. We observed that the numbers of CD11b^+^F4/80^+^ macrophages and CD80^+^ proinflammatory macrophages isolated from the ANGPTL4 group were significantly reduced compared with those isolated from the PBS group (Fig. 2e). Next, we assessed the distribution of macrophages in the plaque lesions by immunohistochemical staining. The area of CD68^+^ macrophages (0.173 ± 0.044 vs. 0.093 ± 0.039 mm^2^, *p* < 0.01) and Mac2^+^ macrophages (0.15 ± 0.041 vs. 0.088 ± 0.03 mm^2^, *p* < 0.01) and the percentage of CD68^+^ macrophages (40.311 ± 5.382% vs. 29.122 ± 5.767%, *p* < 0.01) and Mac2^+^ macrophages (32.59 ± 5.575% vs. 24.92 ± 6.722%, *p* < 0.05) within plaques were decreased in the ANGPTL4 group (Fig. 2f, g). Taken together, these data demonstrate that ANGPTL4 significantly reduced the macrophage inflammatory response and lipid accumulation.

### ANGPTL4 administration attenuates atherogenic mediators in *Apoe−/−* mice

**Fig. 3:**
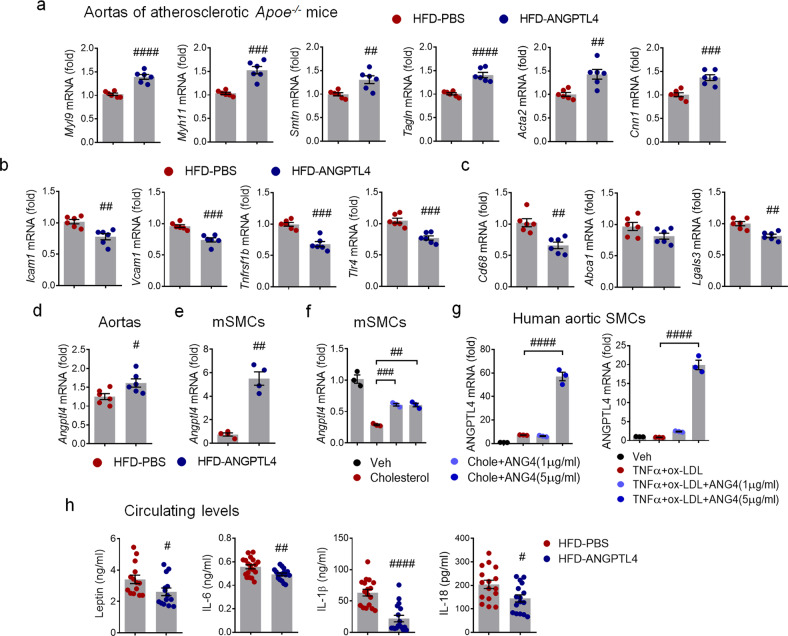
The expression patterns of atherogenic mediators from the aortas and plasma of *Apoe−/−* mice. Relative expression of genes related to contractility (**a**), proinflammation (**b**), and macrophage markers (**c**) in aortas isolated from the PBS and ANGPTL4 groups. *Apoe−/−* mice fed a high-fat diet (HFD) were injected with PBS or ANGPTL4 twice a week for 8 weeks (2 μg, *i.p*.). ANGPTL4 expression in aortas (**d**) and aortic SMCs (**e**, **f**) of *Apoe−/−* mice and human aortic SMCs (**g**). Aortic SMCs were stimulated with cholesterol (10 μg/ml) or TNFα (100 ng/ml) and oxLDL (10 μg/ml) with or without ANGPTL4. **h** Circulating leptin, IL-6, IL-1β, and IL-18 were quantified in the PBS and ANGPTL4 groups. Data were presented as the mean ± SEM. #*p* < 0.05, ##*p* < 0.01, ###*p* < 0.001, ####*p* < 0.0001 (by Student’s *t*-test or one-way ANOVA with Bonferroni’s multiple-comparisons test).

To investigate how ANGPTL4 affects the atherosclerotic response in cells, we studied the mRNA expression in the entire aorta using real-time PCR. The modulation of VSMCs from the contractile phenotype to the synthetic/proliferative phenotype is a critical step in the pathogenesis of vascular diseases^20,21^. Contractile VSMC markers, such as Myh9, Myh11, Smtn, Tagln, Acta2, and Cnn1, were significantly upregulated in the ANGPTL4 group (Fig. 3a). SMCs express cell transition markers^22,23^ and lose the expression of contractile markers in pathological lesions^18^. We found that the mRNA expression of proinflammatory markers such as Icam-1, Vcam-1, Tnfrsf11b, and Tlr4 was significantly lower in the ANGPTL4 group than in the PBS group (Fig. 3b). The expression levels of macrophage markers such as CD68 and Lgals3 were decreased by ANGPTL4 administration (Fig. 3c). Moreover, we compared the ANGPTL4 mRNA levels in aortic tissues and aortic SMCs isolated from atherosclerotic mice administered PBS or ANGPTL4 (Fig. 3d, e). Additionally, the effect of ANGPTL4 treatment on mouse SMCs and human aortic SMCs was analyzed (Fig. 3f, g). Collectively, ANGPTL4 mRNA was markedly increased by ANGPTL4 treatment in aortic tissues and aortic SMCs (Fig. 3d–g). Similarly, we examined the systemic effects of ANGPTL4 administration on circulating inflammatory cytokines in the plasma of *Apoe−/−* mice. Elevated levels of circulating leptin, IL-6, IL-1β, and IL-18 were profoundly reduced in the ANGPTL4 group (Fig. 3h). The circulating ANGPTL4 levels were not significantly different between the two groups (Supplementary Fig. 2). These results demonstrate that ANGPTL4 reduces mediators of vascular inflammation and supports a contractile phenotype in SMCs.

### ANGPTL4 treatment attenuates the proliferation of VSMCs

Because the migration and proliferation of VSMCs contribute to atherosclerosis and cardiovascular disease^24^, we examined the effect of ANGPTL4 treatment on the phenotypes of stimulated VSMCs. We assayed cell proliferation by the BrdU incorporation assay and showed that ANGPTL4 treatment substantially inhibited proliferation in human aortic SMCs stimulated with either FBS or platelet-derived growth factor-BB (PDGF-BB) (Supplementary Fig. 3a). Ki67 staining also showed that ANGPTL4 treatment inhibited proliferation in human aortic SMCs. The numbers of Ki67-positive cells were lower in both PDGF-BB- and FBS-stimulated human aortic SMCs after ANGPTL4 treatment (Supplementary Fig. 3b).

### ANGPTL4 administration stabilizes atherosclerotic plaques

**Fig. 4:**
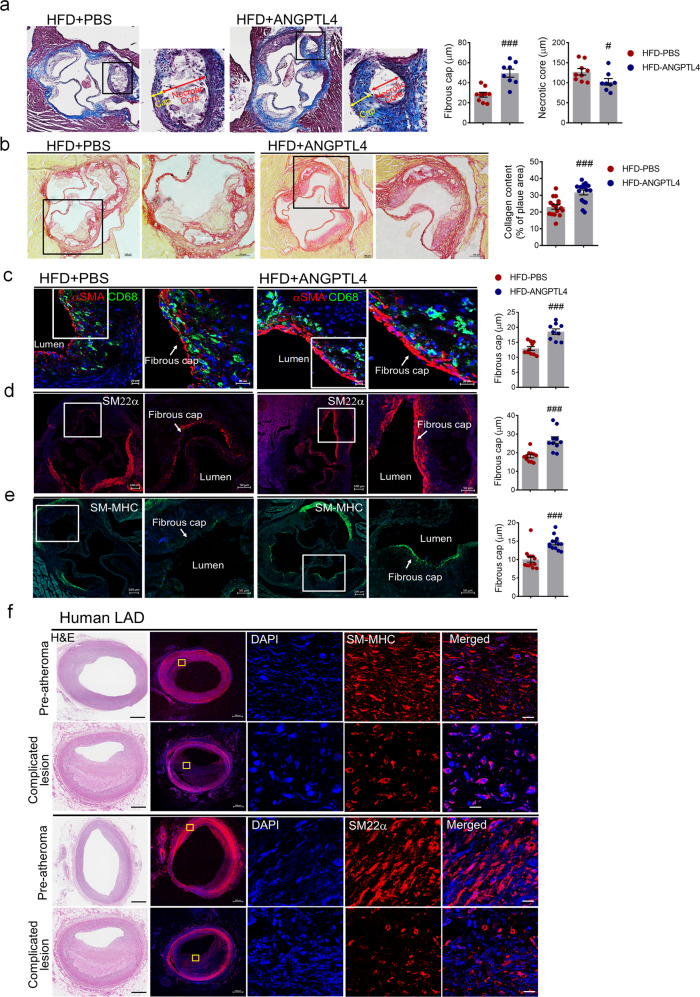
Effects of ANGPTL4 on the stability of atherosclerotic plaques. **a**–**e** Representative histologic analysis of the aortic root from the PBS and ANGPTL4 groups. **a** Representative images of Masson trichrome staining are shown. Boxed areas in the aortic root depict the fibrous cap and necrotic core at higher magnification, and the fibrous cap and necrotic core were measured as the lesion thickness. Scale bar, 100 μm. **b** Representative images of picrosirius red staining for collagen, and quantification of the collagen content presented as a percentage of the plaque area. Scale bar, 100 μm. Immunofluorescence staining of atherosclerotic plaques showed α-SMA and CD68 (**c**), SM22α (**d**), and SM-MHC (**e**) in the fibrous caps. Quantification of the fibrous cap thickness is presented in the right panels. Scale bar, 20 μm. **f** H&E (left panels) and SM-MHC and SM22α-stained confocal images of lesions representing preatheromatous plaques and complicated lesions of the human LAD. Scale bar, 10 μm. Data were presented as the mean ± SEM. #*p* < 0.05, ###*p* < 0.001 (by Student’s *t-*test). LAD left anterior descending artery.

Because systemic ANGPTL4 injection may affect more than the inflammatory status, we further examined whether plaque stability, a key factor during the pathogenesis of atherosclerosis, may be causally involved in the alleviation of atherosclerosis. Most importantly, the measurement of fibrous cap thickness revealed that the atherosclerotic lesions of the ANGPTL4-treated *Apoe−/−* mice had smaller necrotic cores (127.260 ± 23.882 vs. 102.371 ± 23.348 μm, *p* < 0.05) and thicker fibrous caps (28.052 ± 7.164 vs. 49.60 ± 11.506 μm, *p* < 0.001), indicating advanced signs of plaque stability compared with those of the PBS-treated mice (Fig. 4a). Importantly, the fibrous cap thickness was 1.77-fold greater in the lesions of ANGPTL4-treated mice than in those of PBS-treated mice. In an *Ldlr−/−* atherosclerosis model, the fibrous cap of the aortic roots and aorta was also thicker in the ANGPTL4 group than in the PBS group (Supplementary Fig. 4a, b). Because collagen plays a key role in determining plaque stability^25^, we also analyzed collagen deposition in the atherosclerotic plaques using picrosirius red staining. Intraplaque collagen levels were significantly higher in the ANGPTL4 group than in the PBS group in aortic root lesions (31.71 ± 5.297 vs. 23.04 ± 5.886, *p* < 0.001), suggesting that ANGPTL4 can increase the stability of atherosclerotic plaques (Fig. 4b).

Because plaque rupture is inversely correlated with the number of contractile VSMCs^26^, we assessed the VSMC marker proteins α-SMA, Sm22α (transgelin), and SM-MHC in atherosclerotic plaques by immunohistochemical staining (Fig. 4c–e). Compared with that in the PBS group, the fibrous cap of aortic roots with ANGPTL4 had a marked increase in the α-SMA^+^ plaque area (1.43-fold), Sm22α^+^ plaque area (1.49-fold), and SM-MHC^+^ plaque area (1.45-fold), which indicated enhanced features of stability. We next examined SMC (SM-MHC and SM22α) expression in lesions with different degrees of human atherosclerotic disease burden. Immunostaining of serial left anterior descending arteries (LADs) for SM-MHC and SM22α revealed decreased expression of contractile SMC markers in the media and intima of complicated lesions compared with preatheromatous plaques of the human LAD (Fig. 4f). Collectively, these data demonstrate that ANGPTL4 increased the thickness of the fibrous cap in atherosclerotic lesions, contributing to atherosclerotic plaque stability.

### ANGPTL4 regulates SMC phenotypic changes in atherosclerotic lesions

**Fig. 5:**
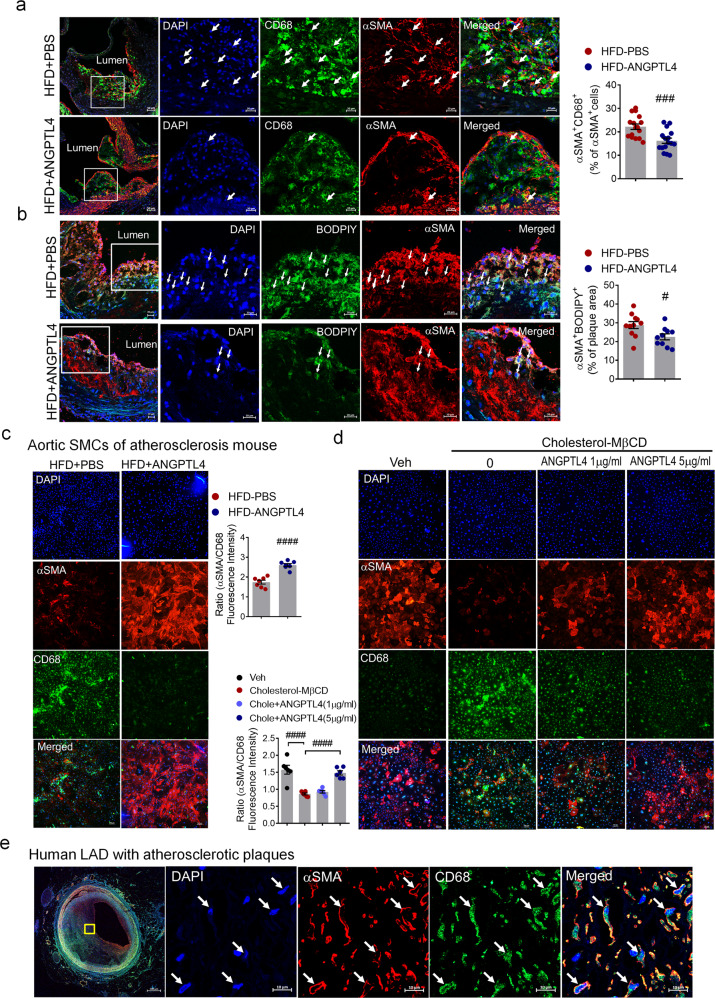
ANGPTL4 regulates SMC phenotypic changes in atherosclerosis. **a** Immunofluorescence staining of atherosclerotic plaques showing CD68 (green) and α-SMA (red) in the aortic root. Boxed areas show close-up images of CD68^+^α-SMA^+^ cells (arrowheads) in atherosclerotic plaques. Quantification of the frequency of double-positive (CD68^+^α-SMA^+^) cells among the total α-SMA^+^ cells within the whole lesion and the fibrous cap (*n* = 16). Scale bar, 20 μm. **b** Representative images of atherosclerotic plaques in the aortic root showing lipid droplets stained by BODIPY (green) and α-SMA (red). Arrowheads indicate BODIPY^+^αSMA^+^ cells. The percentage of BODIPY^+^α-SMA^+^ cells within the plaque area (*n* = 11). Scale bar, 20 μm. **c** Aortic SMCs were isolated from atherosclerotic *Apoe−/*− mice treated with PBS or ANGPTL4 and then stained with α-SMA as an SMC marker and CD68 as a macrophage marker. Quantification of α-SMA/CD68 fluorescence intensity. Scale bar, 100 μm. **d** Aortic SMCs were stimulated with cholesterol (10 μg/ml) for 72 h with or without ANGPTL4 and stained with α-SMA and CD68. Scale bar, 100 μm. **e** In human LAD, the atherosclerotic lesion displayed cells double positive for α-SMA and CD68. Scale bar, 10 μm. Data were presented as the mean ± SEM. #*p* < 0.05, ###*p* < 0.001, ####*p* < 0.0001 (by Student’s *t*-test or one-way ANOVA with Bonferroni’s multiple-comparisons test).

To assess the characteristics of the plaque, we stained the atherosclerotic lesions for macrophages and SMCs. In atherosclerotic *Apoe−/−* mice, ANGPTL4 administration reduced the expression of the macrophage marker CD68 in many α-SMA^+^ cells in the plaque and adjacent media of the atherosclerotic aortic roots compared with PBS administration (Fig. 5a). To test whether ANGPTL4 is directly involved in VSMC-derived foam cell formation, we stained plaques with α-SMA and performed lipid staining with fluorescent BODIPY, which is commonly used to fluorescently stain neutral lipids. The ANGPTL4 group had less lipid deposition in SMC^+^ cells in the plaque relative to the PBS group, suggesting that ANGPTL4 effectively reduces the size of the lipid core and VSMC-derived foam cells in advanced atherosclerosis (Fig. 5b).

In SMCs isolated from atherosclerotic *Apoe-/-* mice, the level of α-SMA was conserved, whereas CD68 was not induced in the ANGPTL4 group (Fig. 5c). SMCs isolated from mouse aortas were stimulated with cyclodextrin–cholesterol complexes with or without ANGPTL4 and then stained with α−SMA and CD68 to confirm their cell type identity (Fig. 5d). Cholesterol-loaded SMCs also showed a significant decrease in α-SMA expression, which was significantly recovered by ANGPTL4 treatment. In contrast, CD68 expression induced by cholesterol was attenuated by ANGPTL4 treatment. Furthermore, ANGPTL4 treatment significantly decreased oxidized LDL uptake and the number of cholesterol-overloaded foam cells among SMCs, indicating that ANGPTL4 reduces lipid accumulation by inhibiting oxidized LDL uptake (Supplementary Fig. 5a, b). As shown in Supplementary Fig. 5c, ANGPTL4 rescued the attenuation of mRNA levels for contractile markers caused by cholesterol in SMCs isolated from mouse aortas. In contrast, the elevated expression of macrophage markers mediated by cholesterol was significantly decreased in ANGPTL4-treated SMCs (Supplementary Fig. 5d). More importantly, cells staining positive for both α-SMA and CD68 were frequently observed in advanced atherosclerotic plaques of human LAD (Fig. 5e). These data indicate that phenotypic changes in SMCs are an ongoing process throughout plaque pathogenesis and that ANGPTL4 plays a key role in regulating VSMC phenotypic modulation.

### ANGPTL4 downregulates KLF4 expression in SMCs in advanced atherosclerotic plaques

**Fig. 6:**
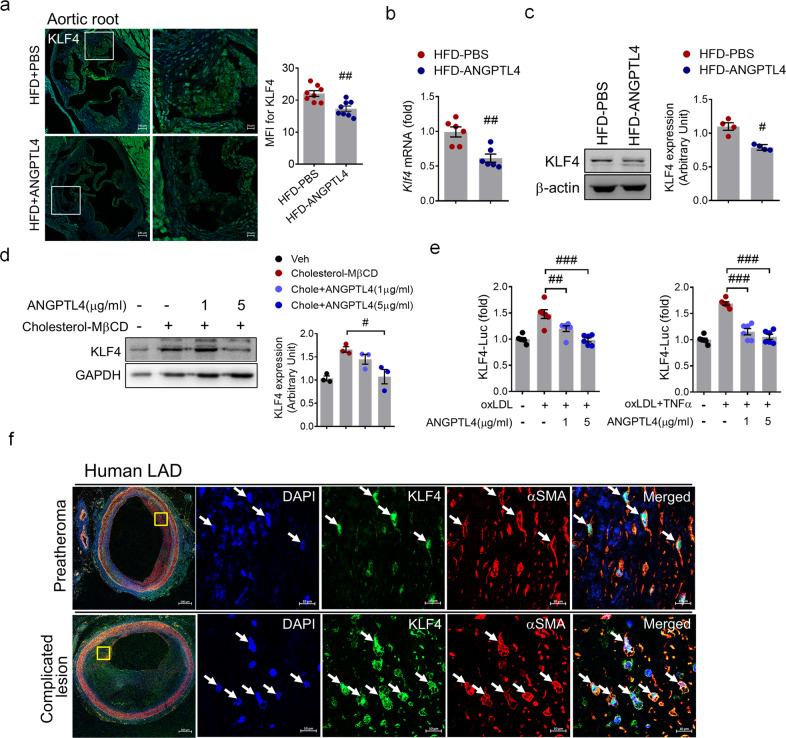
Attenuation of KLF4 upregulation by ANGPTL4 in atherosclerosis. **a** Expression of KLF4 within the plaque of the aortic root from the two groups was determined by immunofluorescent staining. Data were the mean fluorescence intensity (MFI) of KLF4 (mean ± SEM, *n* = 8). Scale bar, 20 μm. **b**, **c** Aortic SMCs were isolated from atherosclerotic *Apoe−/−* mice treated with PBS or ANGPTL4, and the level of KLF4 was measured. The levels of KLF4 mRNA (**b**) and protein (**c**) were lower in the ANGPTL4 group than in the PBS group. **d** Western blotting of KLF4 expression in SMCs from *Apoe−/*− mice pretreated with ANGPTL4 for 24 h and then stimulated with cholesterol (10 μg/ml) for 72 h. **e** KLF4 promoter activity was increased in human aortic SMCs stimulated by oxLDL (10 μg/ml) or oxLDL and TNFα (100 ng/ml) for 24 h but was inhibited by ANGPTL4 (1, 5 μg/ml) treatment. **f** Representative images of α-SMA^+^KLF4^+^ staining in preatheromatous plaques and complicated lesions of the human LAD. Scale bar, 10 μm. Data were presented as the mean ± SEM. #*p* < 0.05, ##*p* < 0.01, ###*p* < 0.001 (by Student’s *t*-test or one-way ANOVA with Bonferroni’s multiple-comparisons test).

KLF4 is a critical transcription factor for the phenotypic changes that occur as VSMCs transition into macrophage-like cells and is upregulated during plaque instability^18^. We investigated whether ANGPTL4 administration downregulates KLF4. We found that KLF4 expression was significantly decreased in both the plaque area and the media layer of the aortic roots in the ANGPTL4 group relative to the PBS group (Fig. 6a). Additionally, KLF4 mRNA and protein in the aorta were also decreased in the ANGPTL4 group compared with the PBS group (Fig. 6b, c). As shown in Fig. 6d and Supplementary Fig. 6a, KLF4 expression was markedly inhibited by ANGPTL4 treatment in cholesterol-stimulated SMCs isolated from *Apoe−/*− mice. Similarly, KLF4 expression was suppressed by treatment with ANGPTL4 in human aortic SMCs and human iPSC-SMCs (Supplementary Fig. 6b). In human aortic SMCs stimulated by TNFα and oxLDL, KLF4 promoter activity was also induced but inhibited by ANGPTL4 treatment (Fig. 6e). Interestingly, KLF4 was substantially upregulated in α-SMA^+^ cells from complicated lesions of human LAD (Fig. 6f). These results indicate that ANGPTL4 downregulates KLF4 expression and plays an important role in regulating VSMC phenotypic changes and plaque instability. Taken together, these data show that ANGPTL4 interferes with plaque development possibly through a reduction in inflammatory factors and cellular changes related to plaque dynamics.

### ANGPTL4 modulates SMC phenotypic changes through KLF4 induction by NOX1

**Fig. 7:**
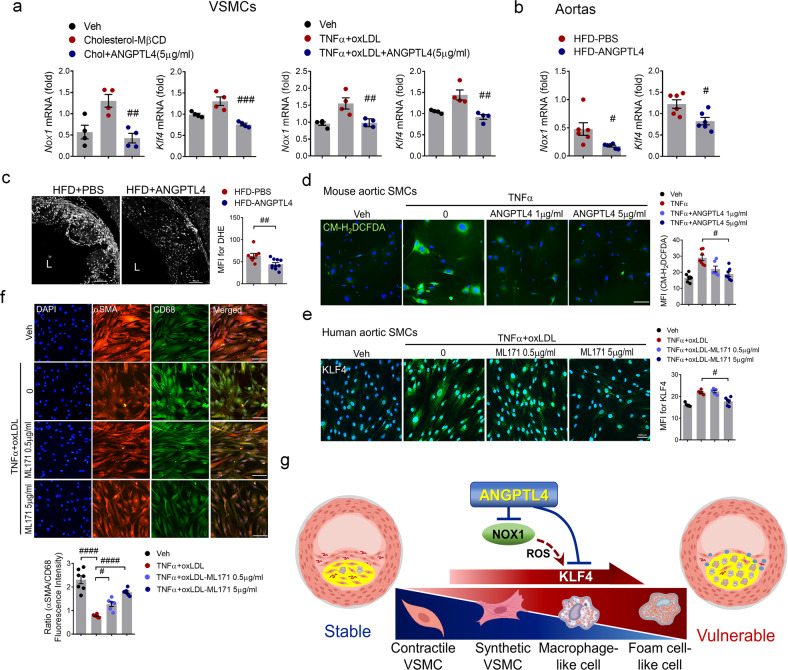
ANGPTL4 modulates SMC phenotypic changes through KLF4 induction by NOX1. **a** Aortic SMCs pretreated with ANGPTL4 were stimulated with cholesterol (10 μg/ml) or oxLDL (10 μg/ml) and TNFα (100 ng/ml), and the levels of Nox1 and KLF4 were measured. **b** Aortas were isolated from atherosclerotic *Apoe−/−* mice treated with PBS or ANGPTL4, and the levels of Nox1 and KLF4 were measured. **c** Representative images and quantification of dihydroethidium (DHE) fluorescence in aortic root sections. The aortic root sections were incubated with 5 μM DHE for 10 min in the dark. The data presented are the MFI of DHE (mean ± SEM, *n* = 9). Scale bar, 100 μm. **d** Representative images of CM-H_2_DCFDA staining. Aortic SMCs from atherosclerotic *Apoe−/−* mice were pretreated with ANGPTL4 and TNFα and exposed to 13 μM CM-H_2_DCFDA. Quantification of ROS levels by MFI. Scale bar, 100 μm. **e**, **f** Human aortic SMCs were stimulated with oxLDL (10 μg/ml) and TNFα (100 ng/ml) with or without ML171, a NOX1 inhibitor (0.5 μg/ml, 5 μg/ml), and were stained with KLF4 (**e**) and α-SMA and CD68 (**f**). Scale bar, 100 μm. **g** Proposed mechanism of action for ANGPTL4 administration in atherosclerosis and plaque stabilization. Data were presented as the mean ± SEM. #*p* < 0.05, ##*p* < 0.01, ###*p* < 0.001, ####*p* < 0.0001 (by Student’s *t*-test or one-way ANOVA with Bonferroni’s multiple-comparisons test).

NOX1-dependent ROS generation is required for VSMC proliferation and migration after vascular injury. Furthermore, the phenotypic change of SMCs to macrophage-like cells is induced by NOXA1-dependent NOX1 activation of KLF4 in atherosclerotic lesions^27^. Thus, we studied whether ANGPTL4 regulates NOX1 activation in KLF4-induced SMC phenotypic changes. The Nox1 gene, but not Nox4 (Supplementary Fig. 7), was induced by cholesterol administration in VSMCs, whereas ANGPTL4 treatment blocked Nox1 induction along with KLF4 (Fig. 7a). Moreover, in an atherosclerosis mouse model, Nox1 and KLF4 were significantly downregulated in the ANGPTL4 group (Fig. 7b). To determine whether the protective effect of ANGPTL4 contributes to reduced ROS, intracellular ROS levels were determined by measuring dihydroethidium (DHE) fluorescence (Fig. 7c) and that of the chloromethyl derivative of dichlorodihydrofluorescein diacetate (CM-H_2_DCFDA), an oxidant-sensitive dye (Fig. 7d). The ROS levels in the aortic sinus atherosclerotic lesions were significantly lower in the ANGPTL4 group than in the PBS group (Fig. 7c). ROS levels determined by CM-H_2_DCFDA fluorescence were increased in aortic SMCs treated with TNF-α. Interestingly, ROS generation was markedly reduced in response to ANGPTL4 (Fig. 7d). Human aortic SMCs were stimulated with TNFα and oxLDL with or without ML171, a pharmacological inhibitor of NOX1, and then stained with KLF4, αSMA, and CD68 to confirm their cell type identity. Notably, KLF4 expression was markedly inhibited by ML171 treatment in human aortic SMCs (Fig. 7e). SMCs stimulated with TNFα and oxLDL showed a significant decrease in α-SMA expression, which was significantly recovered by ML171 treatment. In contrast, the expression of CD68 induced by TNFα and oxLDL was attenuated by ML171 treatment (Fig. 7f). Collectively, these findings suggest that ANGPTL4 is a key regulator of NOX1 activation of KLF4-induced SMC phenotypic changes in atherosclerotic plaques (Fig. 7g).

### Clinical implications of ANGPTL4

Using ELISA, we analyzed the circulating levels of ANGPTL4 in patients with MI (Supplementary Fig. 8a). The clinical characteristics of the two groups are summarized in Table 1. The patients’ mean ages were 63.4 years and 64.9 years in the low and high ANGPTL4 groups, respectively. A total of 79.6 and 76.9% of patients were male in the low and high ANGPTL4 groups, respectively. In the low ANGPTL4 group, 99% of patients were prescribed statins as a discharge medication, and 40.8% of these patients were prescribed ticagrelor or prasugrel. In the high ANGPTL4 group, 95.2% of patients were prescribed a statin, and 51% were prescribed one of the newer P2Y12 inhibitors. The mean ANGPTL4 levels of the low and high ANGPTL4 groups were 0.925 ± 0.42 and 3.09 ± 2.28 ng/mL, respectively. Compared with patients with a lower level of ANGPTL4, patients with a higher level of ANGPTL4 had lower levels of triglycerides (120.28 ± 67.93 vs. 144.00 ± 83.78 mg/dL) and a lower LVEF (53.86 ± 11.19% vs. 59.48 ± 9.79%). The sex proportions and the prevalence of atherosclerotic risk factors, such as hypertension, diabetes mellitus, dyslipidemia, and familial history of coronary artery disease or smoking, were comparable between the two groups. Evidence-based medications for MI, such as antiplatelets, beta-blockers, renin-angiotensin-aldosterone system blockers, and statins, were also similarly prescribed to patients in both groups. Given that normal triglyceride levels are less than 150 mg/mL, the average range of triglycerides in this study was not considered pathological. Reduced TG levels may be a secondary effect of the beneficial changes related to ANGPTL4.

**Table 1:** Baseline clinical characteristics of patients with ANGPTL4 levels above or below the median.

Factor	Low ANGPTL4 (*n* = 103)	High ANGPTL4 (*n* = 104)	*P* value
Age (years)	63.4 ± 12.6	64.9 ± 12.3	0.379
Men, *n* (%)	82 (79.6)	80 (76.9)	0.639
Systolic BP (mmHg)	126.0 ± 22.6	127.0 ± 24.5	0.614
Heart rate (/min)	76.0 ± 17.5	80.9 ± 19.3	0.056
Current or ex-smoker, *n* (%)	69 (67.0)	60 (57.7)	0.168
Hypertension, *n* (%)	55 (53.4)	47 (45.2)	0.238
Diabetes mellitus, *n* (%)	37 (35.9)	27 (26.0)	0.758
Dyslipidemia, *n* (%)	25 (24.3)	18 (17.3)	0.217
Obesity, *n* (%)	36 (35.0)	37 (35.6)	0.925
Familial history of CAD, *n* (%)	11 (10.7)	7 (6.7)	0.313
ANGPTL4 levels (ng/mL)	0.925 ± 0.42	3.09 ± 2.28	**<0.001**
Serum creatinine (mg/dL)	1.96 ± 6.17	1.12 ± 0.96	0.517
Peak troponin-I (mg/dL)	29.11 ± 46.33	41.65 ± 57.09	0.101
Peak CK-MB (mg/dL)	38.97 ± 69.30	61.72 ± 89.53	0.067
Total cholesterol (mg/dL)	176.59 ± 39.74	178.79 ± 43.32	0.712
Triglyceride (mg/dL)	144.00 ± 83.78	120.28 ± 67.93	**0.043**
HDL-cholesterol (mg/dL)	43.00 ± 10.07	42.65 ± 10.908	0.830
LDL-cholesterol (mg/dL)	112.22 ± 31.52	111.86 ± 30.91	0.940
Serum glucose (mg/dL)	145.01 ± 51.81	163.40 ± 90.753	0.547
N-terminal pro BNP (pg/mL)	2,130 ± 3,798.77	3,309.63 ± 7,075.20	0.340
High-sensitivity CRP (mg/L)	1.38 ± 3.31	1.43 ± 3.19	0.924
Platelets (×10^9^/L)	239.74 ± 65.52	273.86 ± 259.582	0.199
Left ventricular EF (%)	59.48 ± 9.79	53.86 ± 11.19	**<0.001**
Medications at discharge, *n* (%)			
Aspirin	103 (100)	104 (100)	1.000
Clopidogrel	61 (59.2)	51 (49.0)	0.141
Prasugrel or ticagrelor	42 (40.8)	53 (51.0)	0.141
Beta-blocker	86 (83.5)	85 (81.7)	0.738
ACEi or ARB	96 (93.2)	89 (85.6)	0.075
Statin	102 (99.0)	99 (95.2)	0.100

Values are presented as the mean ± SD or number (percentage).

*ACEi* angiotensin-converting enzyme inhibitor, *ARB* angiotensin-II receptor blocker, *BNP* brain-type natriuretic peptide, *BP* blood pressure, *CAD* coronary artery disease, *CK-MB* creatine kinase-myocardial band isoenzyme, *CRP* C-reactive protein, *EF* ejection fraction, *HDL* high-density lipoprotein, *LDL* low-density lipoprotein.

Bold values indicates statistically significant *p* values less than 0.05 (<0.05).

**Fig. 8:**
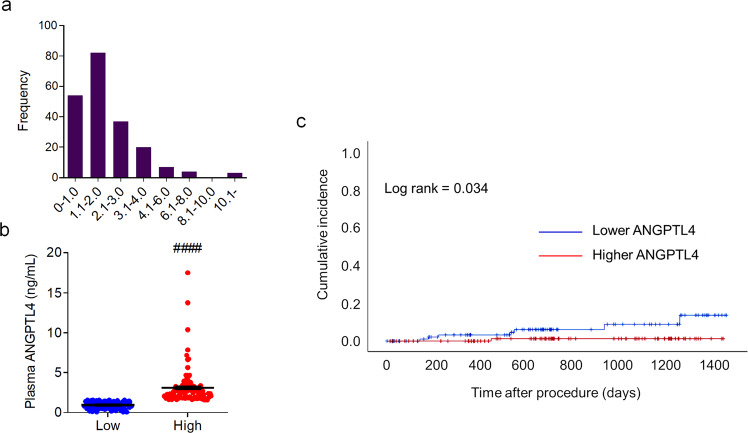
Circulating levels of ANGPTL4 in patients with cardiovascular disease. **a** Distribution of plasma levels of ANGPTL4 measured in all patients (*n* = 207). **b** Plasma levels of ANGPTL4 in the low ANGPTL4 (*n* = 103) and high ANGPTL4 (*n* = 104) groups. **c** Kaplan‒Meier curve illustrating the vascular event incidence of patients during the follow-up period after surgery based on plasma ANGPTL4 levels above (red) and below (blue) the median value. Data were presented as the mean ± SEM. ####*p* < 0.0001 (by Student’s *t*-test).

The distribution of plasma levels of ANGPTL4 ranged from 0.065 to 17.491 ng/mL, and the median value was 1.581 ng/mL (Fig. 8a). Table 2 shows the angiographic and procedural characteristics of patients in the low and high ANGPTL4 groups. There were no significant differences in the proportion of patients with multivessel disease, ACC/AHA lesion complexity, frequencies of pre-PCI TIMI flow 0, post-PCI TIMI flow 3, or symptom-to-balloon time between the groups.

**Table 2:** Coronary angiographic and procedural characteristics of patients with ANGPTL4 levels above or below the median.

Factor	Low ANGPTL4 (*n* = 103)	High ANGPTL4 (*n* = 104)	*P* value
MVD, *n* (%)	48 (46.6)	51 (49.0)	0.726
ACC/AHA B2/C lesion, *n* (%)	100 (97.1)	103 (99.0)	0.369
Pre-PCI TIMI flow grade 0, *n* (%)	39 (37.9)	46 (42.7)	0.352
Symptom-to-balloon time (min)	7,420 ± 18,874	3,956 ± 13,226	0.150
Post-PCI TIMI flow grade 3, *n* (%)	101 (98.1)	101 (97.1)	1.000

Values are presented as the mean ± SD or number (percentage).

*ACC* American College of Cardiology, *AHA* American Heart Association, *MVD* multivessel disease, *PCI* percutaneous coronary intervention, *TIMI* thrombolysis in myocardial infarction.

The median values of plasma ANGPTL4 in the study population were 0.922 (0.065-1.569) ng/mL in the low ANGPTL4 group and 2.548 (1.581–17.491) ng/mL in the high ANGPTL4 group (Fig. 8b). Patients in the high ANGPTL4 group had a higher rate of event-free survival than those in the low ANGPTL4 group (*p* = 0.034 by the log-rank test) (Fig. 8c). Seven patients (6.8%) had a vascular event during follow-up in the low ANGPTL4 group. The rate of vascular events was 9.26 per 100,000 person-years. In the high ANGPTL4 group, only one patient had a vascular event during follow-up, and the rate was 1.22 per 100,000 person-years.

In the multivariate analysis, plasma ANGPTL4 levels were independently associated with an increased risk of future vascular events, and the hazard ratio was 0.185 (95% CI, 0.044 to 0.783; *p* = 0.022). Other factors independently associated with an increased risk of vascular events were a positive family history of CAD and hs-CRP levels at admission (Table 3).

**Table 3:** Factors associated with events of plaque instability.

Factor	Crude hazard ratio on univariate analysis (95% CI)	*P* value	Adjusted hazard ratio on multivariate analysis (95% CI)	*P* value
Age (per year increase)	1.015 (0.955–1.079)	0.630		
Female	1.360 (0.274–6.750)	0.707		
Hypertension	1.421 (0.339–5.959)	0.631		
Diabetes mellitus	1.104 (0.222–5.488)	0.904		
Dyslipidemia	28.650 (0.017–48008.246)	0.376		
Obesity	1.086 (0.259–4.556)	0.910		
Smoking	1.960 (0.448–7.867)	0.343		
Family history of CAD	0.199 (0.047–0.840)	0.028	0.002 (0.000–0.766)	**0.041**
Systolic blood pressure	1.017 (0.988–1.046)	0.253		
Heart rate	0.997 (0.959–1.037)	0.885		
Pain to balloon time	0.941 (0.729–1.213)	0.639		
MVD	0.879 (0.219–3.527)	0.856		
Culprit LAD	3.657 (0.736–18.162)	0.113	512.950 (0.502-524347.409)	0.078
Creatinine	0.785 (0.194–3.183)	0.735		
ANGPTL4 levels	0.422 (0.162–1.101)	0.078	0.199 (0.049-0.808)	**0.024**
Peak CK-MB	0.996 (0.984–1.009)	0.584		
Peak troponin-I	0.973 (0.936–1.012)	0.167	0.863 (0.736-1.013)	0.072
hsCRP	1.195 (1.050–1.359)	0.007	1.178 (1.023-1.356)	**0.023**
Initial LVEF	0.999 (0.992–1.006)	0.830		
Ticagrelor or prasugrel at discharge	1.017 (0.944–1.095)	0.662		
Beta-blocker at discharge	1.878 (0.469–7.517)	0.373		
ACEi or ARB at discharge	1.294 (0.261–6.421)	0.758		
Aldosterone antagonist at discharge	0.041 (0.000–562.583)	0.511		
Use of statin at discharge	0.047 (0.000–3631445.951)	0.742		

*ACEi* angiotensin-converting enzyme inhibitor, *ARB* angiotensin-II receptor blocker, *CAD* coronary artery disease, *CI* confidence interval, *CK-MB* creatine kinase-myocardial band isozyme, *hsCRP* high-sensitivity C-reactive protein, *LAD* left anterior descending, *LVEF* left ventricular ejection fraction, *MVD* multivessel disease.

Bold values indicates statistically significant *p* values less than 0.05 (<0.05).

Furthermore, we analyzed the correlation between ANGPTL4, heart failure, and the incidence of recurrent heart failure (Supplementary Tables 4, 5). The distribution of plasma ANGPTL4 is shown in Supplementary Fig. 8b. The median values of plasma ANGPTL4 in the study population were 1.547 (0.392–2.620) ng/mL in the low ANGPTL4 group and 4.923 (2.651–13.746) ng/mL in the high ANGPTL4 group (Supplementary Fig. 8c). Similarly, plasma levels of ANGPTL4 showed a negative correlation with the incidence of recurrent heart failure (Supplementary Fig. 8d and Supplementary Table 6).

## Discussion

Current guidelines include no other drugs except statins for broad use in atherosclerotic diseases to target plaque vulnerability. However, even with high-dose statin therapy, some patients experience plaque destabilization and subsequent cardiovascular events, such as stroke and AMI. There is indeed an unmet need for antiatherosclerotic therapy beyond lipid lowering.

Human genetics studies of carriers of a missense E40K variant of ANGPTL4 or other inactivating ANGPTL4 mutations show that these carriers have lower levels of triglycerides^28,29^. ANGPTL4, which is regulated by nutritional and other metabolic states in a tissue-dependent manner, regulates many metabolic and nonmetabolic processes^30–32^. The most well-recognized action of ANGPTL4 is the posttranscriptional regulation of lipoprotein lipase, a function that is shared with ANGPTL3 and ANGPTL8^33,34^.

In an atherosclerotic mouse model, transgenic overexpression of ANGPTL4 in *Apoe−/*− mice attenuates atherosclerosis primarily by suppressing lipid uptake in macrophages without changing the plasma levels of cholesterol or triglycerides^35^. Conversely, global ANGPTL4 deficiency in *Ldlr*−/− mice reduces atherosclerosis, whereas hematopoietic cell-specific ANGPTL4 deletion results in larger atherosclerotic plaques with enhanced foam cell formation^36^.

We recently identified that ANGPTL4 is copiously released from mesenchymal stem cells in response to inflammatory stimuli such as IL-1β, TNF-α, and inflammatory macrophages. In a mouse MI model, ANGPTL4 administration significantly reduces cardiac injury as well as systemic and cardiac inflammation^16^. To further develop clinical-translational approaches, we investigated the clinical characteristics of ANGPTL4 and validated the therapeutic efficacy of ANGPTL4 administration in an atherosclerosis mouse model. Chronic hyperlipidemia may initiate endothelial injury, resulting in endothelial permeability, enhanced leukocyte adhesion, and alterations in the expression of adhesive molecules. Vascular inflammation and plaque stability are determinative factors that contribute to the accelerated development of atherosclerotic lesions. VSMCs are key cellular components of arteries, exhibit phenotypic plasticity, and can switch from a contractile phenotype to a synthetic or proliferating phenotype in response to extracellular stimuli. In particular, the contractile phenotype of VSMCs with high expression of transgelin actively participates in suppressing NF-κB inducing kinase (NIK)-involved inflammation in VSMCs^37^. In our study, VSMCs underwent phenotypic modulation, and ANGPTL4-treated *Apoe−/−* mice showed a remarkable increase in the expression of genes related to the contractile phenotype in aortas.

Vascular stability is determined by macrophage-secreted proteases, VSMC phenotype, and the synthesis of an elastin-rich extracellular matrix. We found that plaques in ANGPTL4-treated mice had a thicker fibrous cap and a markedly smaller necrotic core. Notably, the latter traits are primary features of stable atherosclerotic plaques, which may suggest that ANGPTL4 reduces plaque size and promotes plaque stability. The fibrous cap, which comprises mostly contractile VSMCs and fibroblasts, is critical for stabilizing and protecting atherosclerotic plaques from rupture, which is a major cause of the clinical sequelae of atherosclerosis. Identification of the mediators governing lesion characteristics would allow the development of specific therapies to stabilize the atherosclerotic plaque, other than those therapies that generally suppress inflammation.

Indeed, VSMCs contribute to 30 to 70% of cells expressing macrophage markers^38^ and lose the expression of contractile proteins such as α-SMA while expressing markers of other cell types such as CD68^39^. Finding a new therapy that attenuates SMC transition and maintains a contractile phenotype could be promising for treating atherosclerotic vascular diseases. SMC phenotypic switching is a key phenomenon underlying several vessel-narrowing diseases, such as atherosclerosis, and our study showed that ANGPTL4 could be a potential regulator of SMC differentiation by blocking the transition to macrophage-like cell types by downregulating KLF4.

In response to pathological conditions associated with inflammation, NOX1 plays a critical role in VSMC function^40,41^. As a supporting mechanism, NOXA1-dependent NADPH oxidase activity in SMCs has been shown to be highly correlated with SMC proliferation and migration, KLF4-mediated transition to a macrophage-like phenotype and plaque inflammation^27^. In cancer, ANGPTL4 is associated with the upregulation of NOX4^42^ and NOX2^43^ expression. In particular, one study described the stimulation of oncogenic ROS and anoikis resistance by ANGPTL4 through activation of NADPH oxidase^44^. We found that ANGPTL4 was responsible for the reduction in TNFα-induced NOX1, the major source of ROS. The anti-inflammatory effect of ANGPTL4 appears to be involved in suppressing the inflammatory responses of NOX-derived ROS that contribute to accelerated atherosclerosis. In summary, our results provide a novel role for ANGPTL4 as a strong negative regulator of NOX1/KLF4-mediated VSMC phenotypic switching.

To support our findings, we then analyzed the relationship between ANGPTL4 levels and clinical outcomes in patients with AMI and heart failure. Patients with high levels of ANGPTL4 showed a lower incidence of recurrent heart failure during follow-up. In the multivariate analysis, a family history of CAD was found to be a protective factor for future vascular events, as previously reported^45^. We also confirmed that the inflammation biomarker hs-CRP is an independent predictor of future vascular events. This is in line with previous studies^46,47^. In addition, MI patients with no-reflow had lower levels of ANGPTL4 than did patients without no-reflow (3.13 ± 4.85 vs. 11.09 ± 9.94 ng/mL; *p* = 0.03)^48^.

The genetically inactive ANGPTL4 variant, which does not inhibit lipoprotein lipase activity, was associated with lower levels of triglycerides and a lower risk of CAD in large-scale DNA sequencing and genetics studies^29,46^. In contrast, ANGPTL4 levels showed no correlation with plasma triglycerides. Plasma ANGPTL4 levels were significantly correlated with age and negatively correlated with HDL-cholesterol but showed no correlation with plasma triglyceride levels. The median ANGPTL4 level was 7.7 (3.2 to 232.4) ng/mL, and the triglyceride and total cholesterol levels were 158.54 ± 84.14 ng/mL and 221.58 ± 39.44 ng/mL, respectively^49^. Interestingly, the triglyceride-lowering E40K variant of ANGPTL4 did not influence plasma ANGPTL4 levels^49,50^. In patients with MI, the serum levels of ANGPTL4 on admission were 7.2 ± 8.8 ng/mL and were not significantly associated with hypercholesterolemia, diabetes, or angiographic variables^48^. Therefore, beneficial effects such as triglyceride lowering may be associated with ANGPTL4 activity, not with the plasma level of ANGPTL4.

Therapeutic approaches targeting inflammation are tasked with improving the clinical outcomes of cardiovascular disease. Despite the beneficial effects of corticosteroids in clinical trials, the risk-benefit ratio seems inconclusive owing to a higher incidence of cardiac rupture with impaired wound healing^51^. Treatment with nonsteroidal anti-inflammatory drugs is also not recommended after MI, as it could result in an increased risk of bleeding and excess thrombotic events^52^. The TNF-α antagonist etanercept appears to be damaging to patients with AMI because it increases platelet activation, although it reduces systemic inflammation to some extent^53^. Etanercept also failed to improve clinical outcomes in patients with congestive heart failure^54^. Compelling evidence of a role for IL-1β signaling in cardiovascular disease has also been presented. The MRC-ILA Heart Study showed that administration of anakinra, a recombinant IL-1 receptor antagonist, increases major adverse cardiovascular events, including myocardial infarction, and suggested that inhibition of IL-1 signaling increases the risk of cardiovascular events^55^. Moreover, IL-1β antibody treatment decreases plaque stability indices in *Apoe−/−* mice with advanced atherosclerosis, indicating an unexpected role of IL-1β in regulating fibrous cap formation^56^.

In our study, ANGPTL4 administration profoundly decreased vascular inflammation and the progression of atherosclerotic plaques and regulated the phenotypic fate of smooth muscle cells into macrophage-like cells, suggesting a protective role in stabilizing atherosclerotic plaques by downregulating the NOX1 activation of KLF4. More strikingly, we showed that the ANGPTL4 plasma levels of AMI patients during PCI were associated with future events caused by plaque instability. Altogether, therapeutic and preventive interventions capable of inducing ANGPTL4 expression may lessen plaque progression in the context of post-MI remodeling and its complications.

## Supplementary information

Supplementary Information
