# Supplementary material for: ANGPTL4 stabilizes atherosclerotic plaques and modulates the phenotypic transition of vascular smooth muscle cells through KLF4 downregulation
Source: Exp Mol Med. 2023 Feb 13;55(2):426–42. doi: 10.1038/s12276-023-00937-x (PMC9981608; doi:10.1038/s12276-023-00937-x)

Supplementary Information

ANGPTL4 stabilizes atherosclerotic plaques and modulates phenotypic transition of vascular smooth muscle cells through KLF4 downregulation

Dong Im Cho[1,7*], Min Joo Ahn[2*], Hyang Hee Cho[1,7], Meeyoung Cho[1,7], Ju Hee Jun[1,7], Bo Gyeong Kang[1,7], Soo yeon Lim[1,7], Soo Ji Yoo[1,7], Mi Ra Kim[1], Hyung-Seok Kim[3], Su-Jin Lee[3], Le Thanh Dat[4], Changho Lee[4,5], Yong Sook Kim[1,6#], Youngkeun Ahn[1,2,7#]

[1]Cell Regeneration Research Center, [2]Department of Cardiology, Chonnam National University Hospital [3]Department of Forensic Medicine, Chonnam National University Medical School, [4]Department of Artificial Intelligence Convergence, Chonnam National University, [5]Department of Nuclear Medicine, Chonnam National University Medical School & Hwasun Hospital, [6]Biomedical Research Institute, Chonnam National University Hospital, [7]Department of Cardiology, Chonnam National University Medical School, Gwangju, Republic of Korea

[*] Authors contributed equally

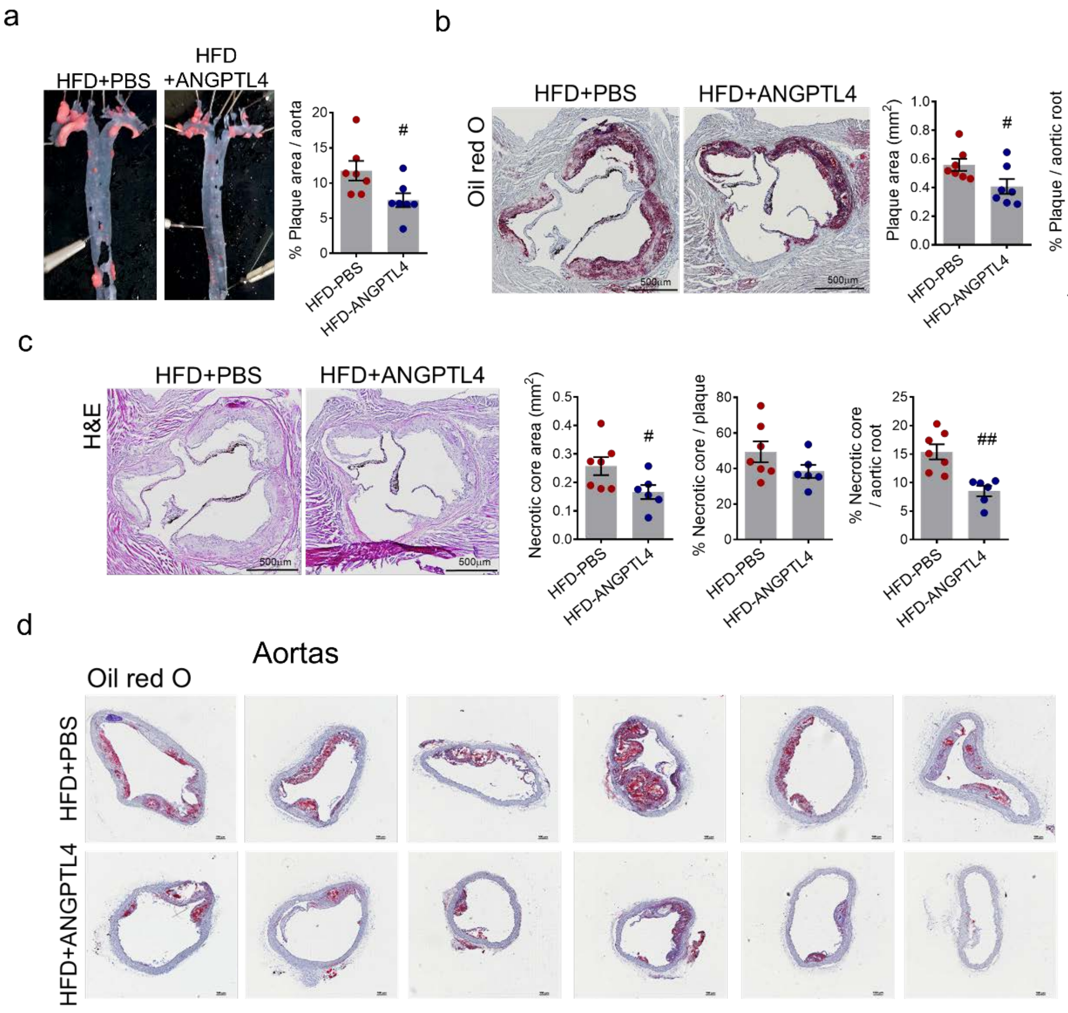
a
HFD+PBS
HFD +ANGPTL4
% Plaque area / aorta
HFD-PBS
HFD-ANGPTL4
b
Oil red O
HFD+PBS
HFD+ANGPTL4
Plaque area (mm2)
% Plaque / aortic root
c
H&E
Necrotic core area (mm2)
% Necrotic core / plaque
% Necrotic core / aortic root
d
Aortas
Oil red O
HFD+PBS
HFD+ANGPTL4

Supplementary Fig 1. The effect of ANGPTL4 administration was examined in a high-fat diet (HFD)-fed *Ldlr-/-* mouse model.

Ldlr-/- mice were fed a HFD with injection of PBS or recombinant ANGPTL4 protein (2 μg per mouse, intraperitoneally twice a week) for 16 weeks, and atherosclerotic plaque area was quantified. **a,** Representative Oil-red O-stained aortas from PBS-injected and ANGPTL4-injected *Ldlr-/-* mice and atherosclerotic plaque area were quantified. **b,** Lesion area measured in Oil-red O-stained cross-sections of the aortic roots from the PBS group and ANGPTL4 group. Plaque area was measured and quantified as the relative size of plaque to the aortic area. Scale bar, 500 μm. **c,** Representative images are shown for H&E-stained aortic root from the PBS and ANGPTL4 groups. Necrotic core area, qualified by anucleated area, was measured and quantified as the relative size of the necrotic core to plaque or aortic root. Scale bar, 500 μm. **d,** Representative images are shown for Oil-red O-stained aortas from the PBS and ANGPTL4 groups. Scale bar, 100 μm. Data are represented as mean ± SEM. #$p < 0.05$, ##$p < 0.01$, ###$p < 0.001$ (by Student's *t*-test).

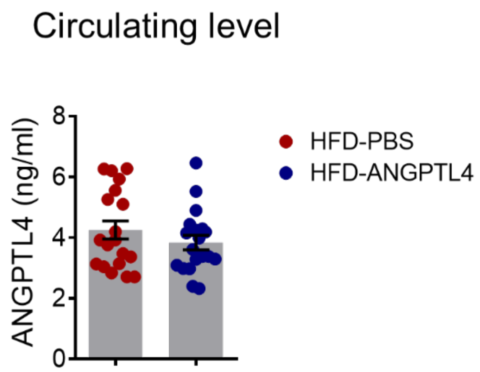

Supplementary Fig. 2. ANGPTL4 expression from plasma of *Apoe-/-* mice.

Circulating ANGPTL4 was quantified in the PBS and ANGPTL4 groups.

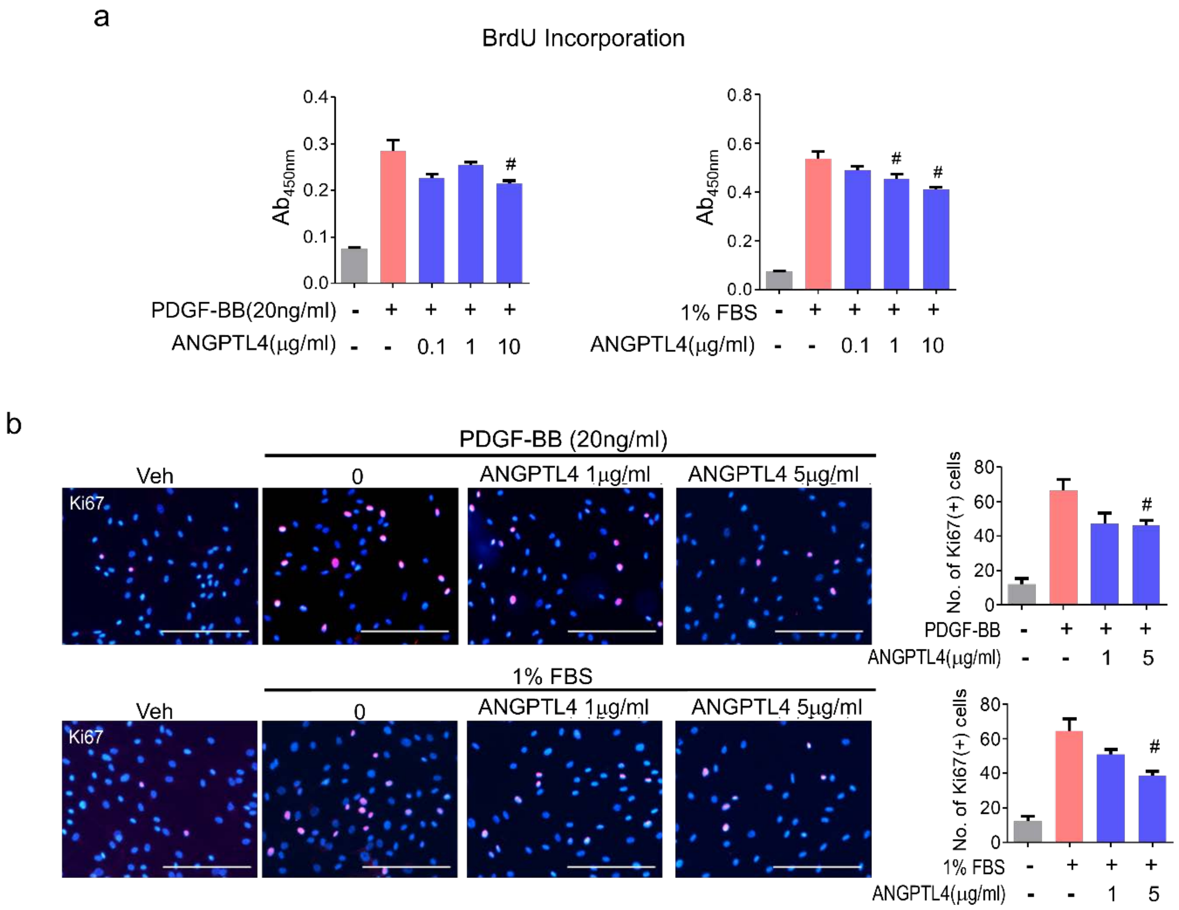

Supplementary Fig 3. The effect of ANGPTL4 on proliferation in human aortic SMCs.

a, Proliferation assay using a measurement of BrdU incorporation in human aortic SMCs. Cell proliferation was initiated with platelet-derived growth factor PDGF-BB (20 ng/mL) or 1% FBS in the presence of ANGPTL4 (0.1, 1, or 10 μg/mL). **b,** Cell proliferation marker Ki67-positive cells were counted in human aortic SMCs stimulated with PDGF-BB or 1% FBS in the presence of ANGPTL4 (1, or 5 μg/mL). Scale bar, 200 μm. Data are represented as mean ± SEM. #p < 0.05 (by one-way ANOVA with Bonferroni's multiple-comparisons test).

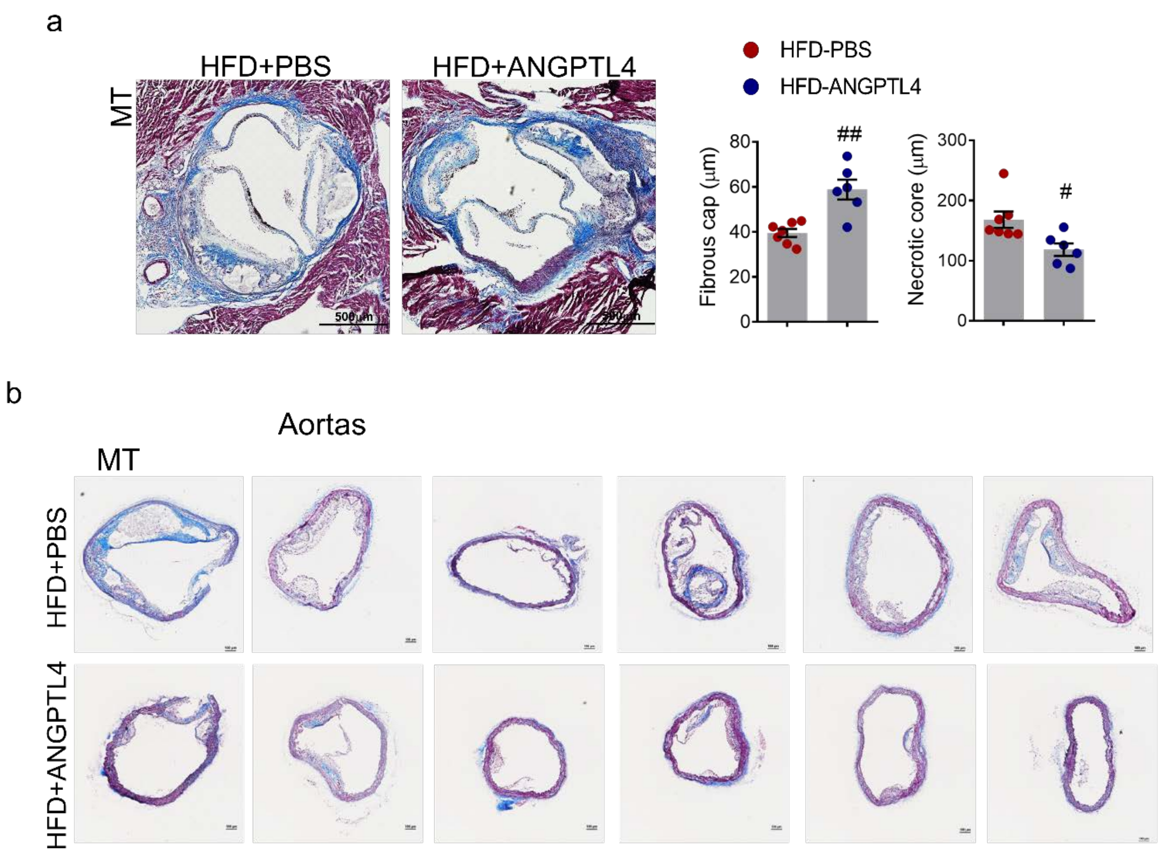

Supplementary Fig 4. Effects of ANGPTL4 on the stability of atherosclerotic plaque in the HFD-fed *Ldlr-/-* mouse model.

a, The thickness of the fibrous cap and the necrotic core in the *Ldlr-/-* mouse atherosclerosis model was measured from Masson trichrome staining. Scale bar, 500 μm. **b,** Representative images are shown for Masson trichrome-stained aortas from the PBS and ANGPTL4 groups. Scale bar, 100 μm. Data are represented as mean ± SEM. #*p*< 0.05, ##*p* < 0.001 (by Student's *t*-test).

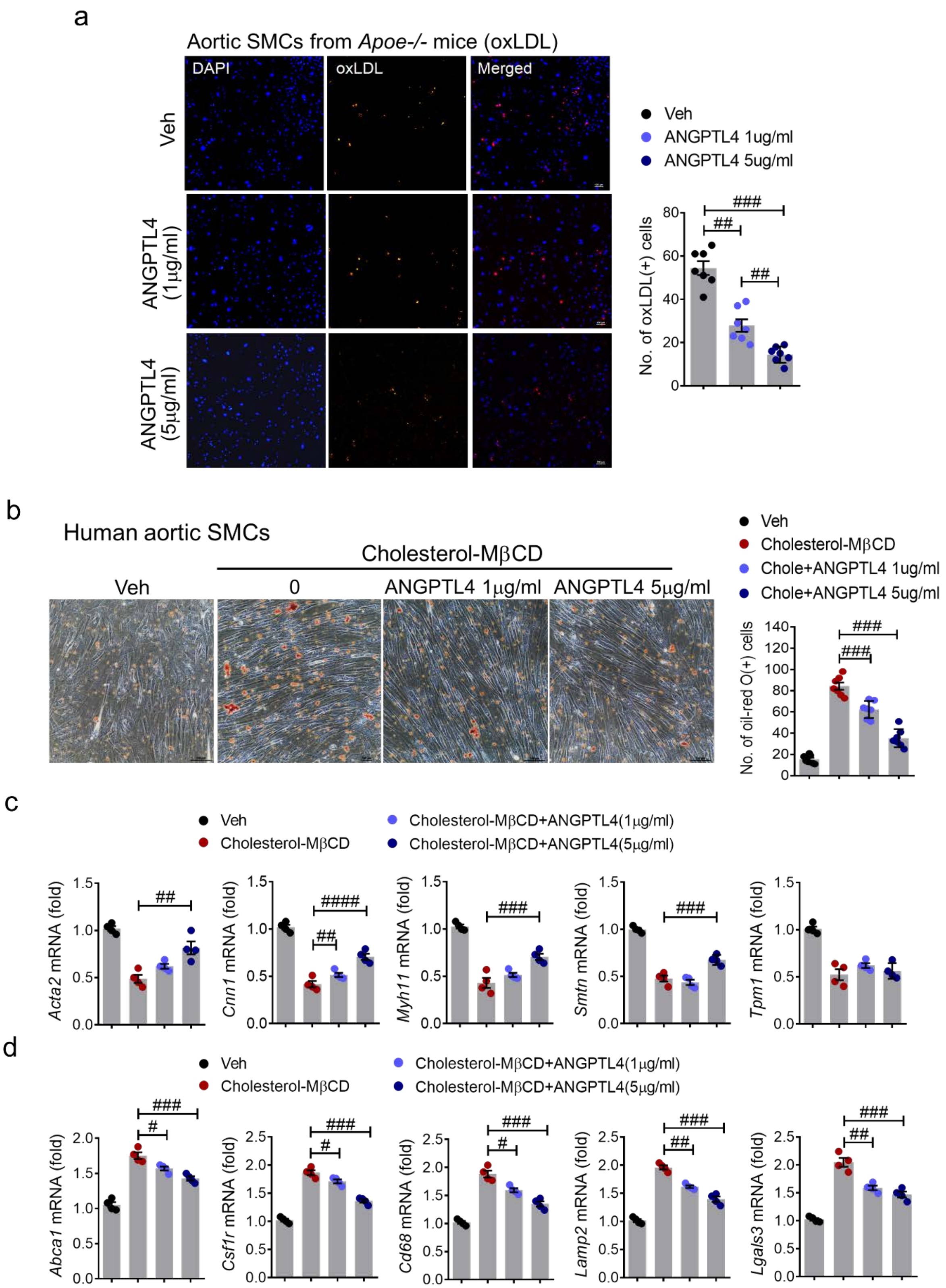

a
Aortic SMCs from Apoe-/- mice (oxLDL)
DAPI
oxLDL
Merged
Veh
ANGPTL4 (1µg/ml)
ANGPTL4 (5µg/ml)
Veh
ANGPTL4 1ug/ml
ANGPTL4 5ug/ml
No. of oxLDL(+) cells
0
20
40
60
80
###
##
##
b
Human aortic SMCs
Cholesterol-MβCD
Veh
0
ANGPTL4 1µg/ml
ANGPTL4 5µg/ml
Veh
Cholesterol-MβCD
Chole+ANGPTL4 1ug/ml
Chole+ANGPTL4 5ug/ml
No. of oil-red O(+) cells
0
20
40
60
80
100
120
140
###
###
c
Veh
Cholesterol-MβCD
Cholesterol-MβCD+ANGPTL4(1µg/ml)
Cholesterol-MβCD+ANGPTL4(5µg/ml)
Acta2 mRNA (fold)
Cnn1 mRNA (fold)
Myh11 mRNA (fold)
Smtn mRNA (fold)
Tpm1 mRNA (fold)
0.0
0.5
1.0
1.5
##
####
##
###
###
d
Veh
Cholesterol-MβCD
Cholesterol-MβCD+ANGPTL4(1µg/ml)
Cholesterol-MβCD+ANGPTL4(5µg/ml)
Abca1 mRNA (fold)
Csf1r mRNA (fold)
Cd68 mRNA (fold)
Lamp2 mRNA (fold)
Lgals3 mRNA (fold)
0.0
0.5
1.0
1.5
2.0
2.5
###
#
###
#
###
#
###
##
###
##

Supplementary Fig 5. The effect of ANGPTL4 treatment on lipid uptake and phenotypic change of SMCs.

a, Aortic SMCs isolated from *Apoe-/-* mice were treated with DiI-oxLDL (10 μg/ml) with or without ANGPTL4 for 24 h and then oxLDL uptake was analyzed. Scale bar, 100 μm. **b,** Human aortic SMCs with cholesterol with or without ANGPTL4 were analyzed using Oil-red O staining. Scale bar, 100 μm. Mouse aortic SMCs were stimulated with cholesterol (10 μg/ml) with or without ANGPTL4, and the relative expression of genes related to contractile **(c)** and macrophage **(d)** markers was analyzed by real-time PCR. Data are represented as mean ± SEM. #p < 0.05, ##p < 0.01, ###p < 0.001, ####p < 0.0001 (by one-way ANOVA with Bonferroni's multiple-comparisons test).

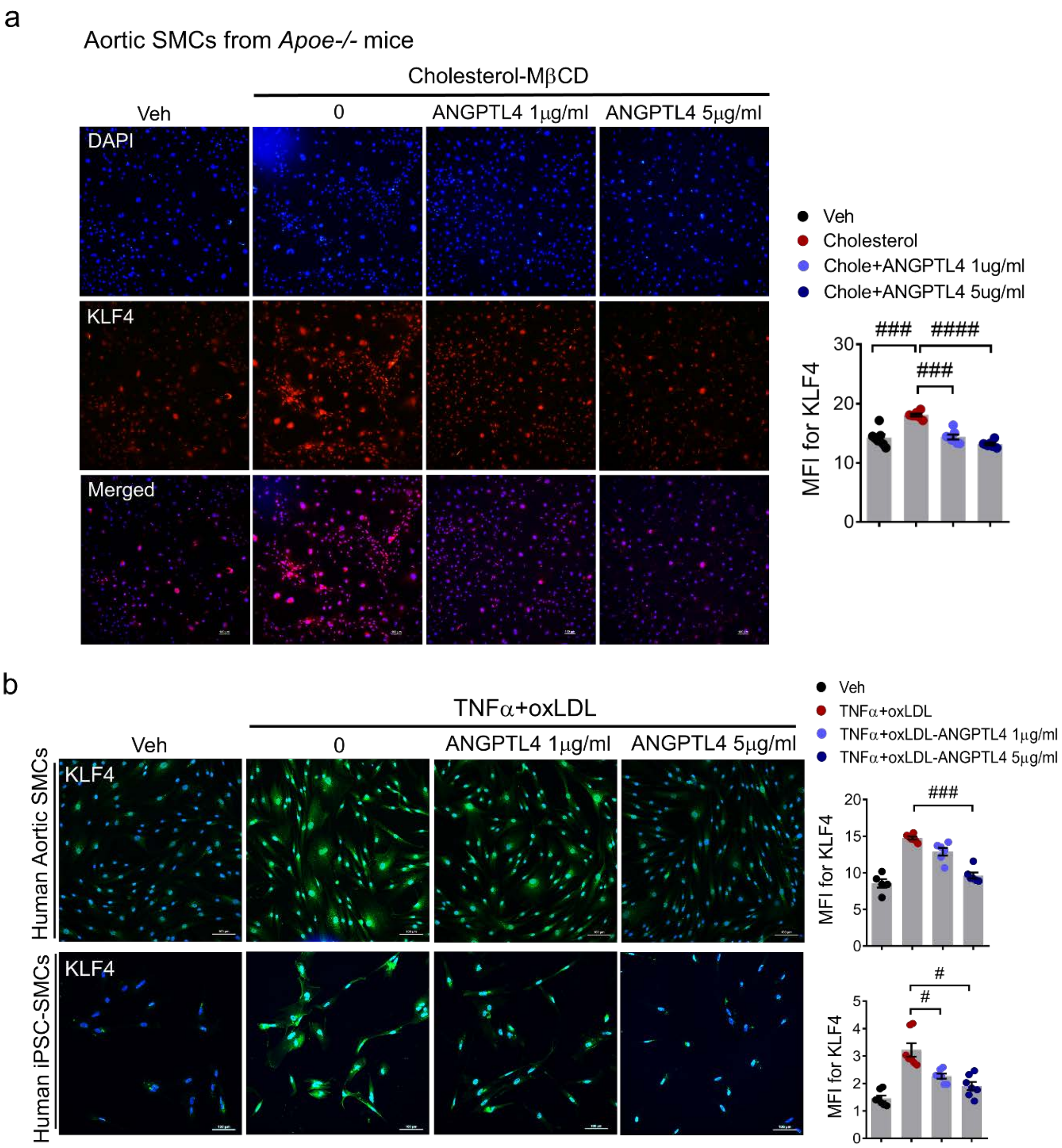
a
Aortic SMCs from Apoe-/- mice
Cholesterol-MβCD
Veh
0
ANGPTL4 1μg/ml
ANGPTL4 5μg/ml
DAPI
KLF4
Merged
Veh
Cholesterol
Chole+ANGPTL4 1ug/ml
Chole+ANGPTL4 5ug/ml
MFI for KLF4
###
####
###
b
TNFα+oxLDL
Veh
0
ANGPTL4 1μg/ml
ANGPTL4 5μg/ml
Human Aortic SMCs
Human iPSC-SMCs
KLF4
KLF4
Veh
TNFα+oxLDL
TNFα+oxLDL-ANGPTL4 1μg/ml
TNFα+oxLDL-ANGPTL4 5μg/ml
MFI for KLF4
###
MFI for KLF4
#
#

Supplementary Fig 6. Immunofluorescence staining of KLF4 expression in SMCs.

a, Aortic SMCs isolated from *Apoe-/-* mice were pretreated with ANGPTL4 for 24 h and then stimulated with cholesterol (10 μg/ml) for 72 h and stained with KLF4. Mean fluorescence intensity of KLF4 was quantified. Scale bar, 100 μm. **b,** Human aortic SMCs and iPSC-SMCs were stimulated with oxLDL (10 μg/ml) and TNFα (100 ng/ml) with or without ANGPTL4 (1 μg/ml, 5 μg/ml) and stained with KLF4. Scale bar, 100 μm. Data are represented as mean ± SEM. #p<0.05, ###$p < 0.001$, ####$p < 0.0001$ (by one-way ANOVA with Bonferroni's multiple-comparisons test).

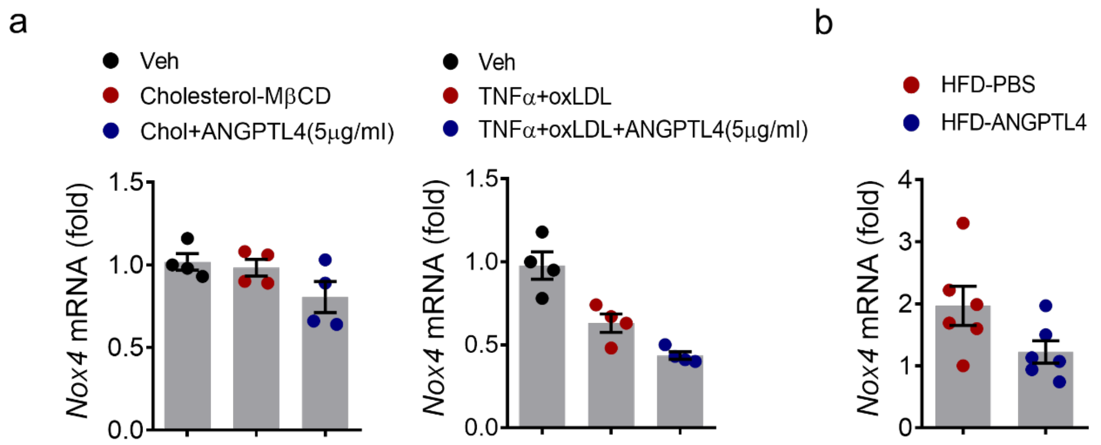

Supplementary Fig 7. Expression patterns of NOX4 in SMCs and aortas of *Apoe-/-* mice.

a, Aortic SMCs from atherosclerotic *Apoe-/-* mice were pretreated with ANGPTL4 and cholesterol (10 μg/ml) or oxLDL (10 μg/ml) and TNFα (100 ng/ml) and the level of Nox4 was measured. **b**, Aortas were isolated from atherosclerotic *Apoe-/-* mice treated with PBS or ANGPTL4, and the level of NOX4 was measured.

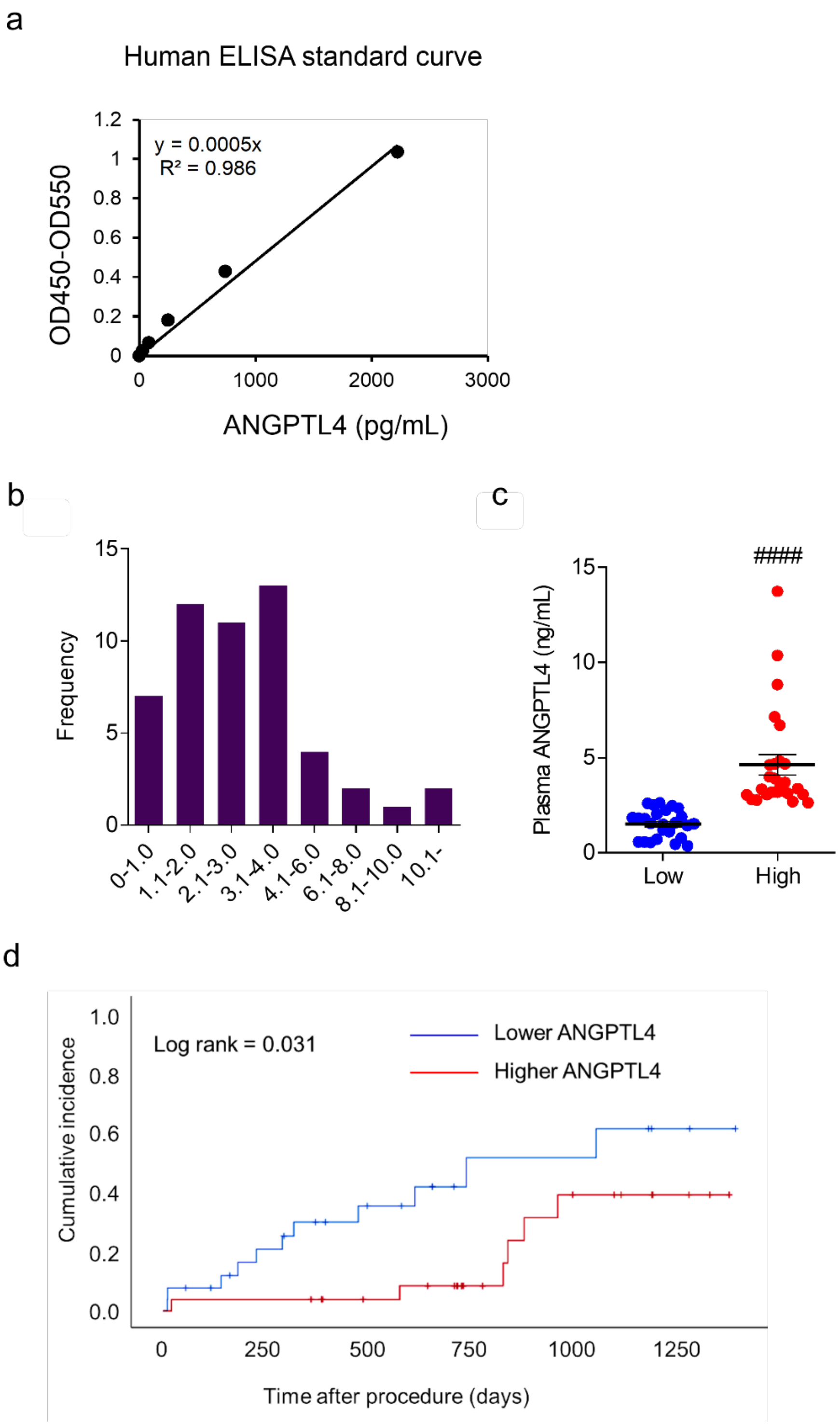
a
Human ELISA standard curve
y = 0.0005x
R² = 0.986
OD450-OD550
1.2
1
0.8
0.6
0.4
0.2
0
0
1000
2000
3000
ANGPTL4 (pg/mL)
b
Frequency
15
10
5
0
0-1.0
1.1-2.0
2.1-3.0
3.1-4.0
4.1-6.0
6.1-8.0
8.1-10.0
10.1-
c
####
Plasma ANGPTL4 (ng/mL)
15
10
5
0
Low
High
d
Cumulative incidence
1.0
0.8
0.6
0.4
0.2
0.0
Log rank = 0.031
Lower ANGPTL4
Higher ANGPTL4
0
250
500
750
1000
1250
Time after procedure (days)

Supplementary Fig 8. Association of circulating ANGPTL4 levels with the incidence of heart failure. **a,** Standard curve for human ANGPTL4 ELISA. **b,** Distribution of plasma levels of ANGPTL4 measured in all patients (n=52). **c,** Plasma levels of ANGPTL4 in the Low ANGPTL4 (n=26) and high ANGPTL4 (n=26) groups. **d,** Kaplan-Meier curve illustrating vascular event incidence of patients during the follow-up period after surgery, based on plasma ANGPTL4 levels above (red) and below (blue) the median value. Data are represented as mean ± SEM. #### $p < 0.0001$ (by Student's *t*-test).

Supplementary Table 1. List of real-time PCR primers

Gene	Primer
Mouse *Angptl4*	Forward : 5’-GGACCTTAACTGTGCCAAGA-3’<br>Reverse: 5’-CGTGGGATAGAGTGGAAGTATTG-3’
Mouse *Gapdh*	Forward : 5’-GGGTGTGAACCACGAGAAATA-3’<br>Reverse: 5’-GTCATGAGCCCTTCCACAAT-3’
Mouse *Tnfrsf11b*	Forward: 5’-GCCGAGAGTGTAGAGAGGATAA-3’<br>Reverse: 5’-CTTCACCATTTCCTGGTCTCTG-3’
Mouse *Tlr4*	Forward: 5’-GCTTACACCACCTCTCAAACT-3’<br>Reverse: 5’-ACAGCCACCAGATTCTCTAAAC-3’
Mouse *Icam1*	Forward: 5’-CTGTTTGAGCTGAGCGAGAT-3’<br>Reverse: 5’-AACGAATACACGGTGATGGTAG-3’
Mouse *Vcam1*	Forward : 5’-CACAGCTCAGTGGACTGGAA-3’<br>Reverse: 5’-CCACCACCACACACCATAGA-3’
Mouse *Il10*	Forward: 5’-ACAGCCGGGAAGACAATAAC-3’<br>Reverse: 5’-CAGCTGGTCCTTTGTTTGAAAG-3’
Mouse *Myh11*	Forward: 5’-GATGAAGAGATCGCCCAGAAA-3’<br>Reverse: 5’-CTCTGAGTCTAGGTCCTCTTGT-3’
Mouse *Smtn*	Forward: 5’-GAGAACTGGCTACACTCTCAAC-3’<br>Reverse: 5’-GTCAACTCCTCGACATCATTCA-3’
Mouse *Tagln*	Forward: 5’-GGAACAGGTGGCTCAATTCT-3’<br>Reverse: 5’-GCTGCCATATCCTTACCTTCAT-3’
Mouse *Myl9*	Forward: 5’-CAGAACCGAGATGGCTTCATT-3’<br>Reverse: 5’-GTTCATCATGCCCTCCAGATAC-3’
Mouse *Klf4*	Forward: 5’- GACCTCCTGGACCTAGACTTTA-3’<br>Reverse: 5’- GAAGACGAGGATGAAGCTGAC-3’
Mouse *Arg1*	Forward: 5’-GATTATCGGAGCGCCTTTCT-3’<br>Reverse: 5’-AAGAATGGAAGAGTCAGTGTGG-3’
Mouse *Nos2*	Forward: 5’- GGAATCTTGGAGCGAGTTGT-3’<br>Reverse: 5’- CCTCTTGTCTTTGACCCAGTAG -3’
Mouse *Ccl2*	Forward: 5’- GGAATCTTGGAGCGAGTTGT-3’<br>Reverse: 5’- CCTCTTGTCTTTGACCCAGTAG -3’
Mouse *Acta2*	Forward: 5’- TCAGGGAGTAATGGTTGGAATG-3’<br>Reverse: 5’- GGTGATGATGCCGTGTTCTA -3’
Mouse *Cnn1*	Forward: 5’- TTGAGAGAAGGCAGGAACATC-3’<br>Reverse: 5’- GTACCCAGTTTGGGATCATAGAG -3’
Mouse *Cd68*	Forward: 5’-GACCCACAACTGTCACTCATAA -3’<br>Reverse: 5’-CTTCCACCCTGAATTGGGTATAG-3’
Mouse *Abca1*	Forward: 5’-GGGTGGTGTTCTTCCTCATTAC-3’<br>Reverse: 5’- CACATCCTCATCCTCGTCATTC-3’
Mouse *Lgals3*	Forward: 5’- ACACGAAGCAGGACAATAACT-3’<br>Reverse: 5’- CAGCTTCAACCAGGACTTGTA-3’

Mouse *Lamp2*	Forward: 5’- TTCAACACCCACTCCAACTC-3’<br>Reverse: 5’- TAGCCAGCAGACAGGTAGTAT-3’
Mouse *Csf1r*	Forward: 5’- CTGGGACAGCACGAGAATATAG-3’<br>Reverse: 5’- CCTTCGGAGAAAGTTGAGTAGG-3’
Mouse *Tpm1*	Forward: 5’- GAGAGTGAGAGAGGCATGAAAG-3’<br>Reverse: 5’- CATACTTCCGGTCAGCATCTT-3’
Mouse *Nox1*	Forward: 5’- CGAGGCTTTAGCATGGAAGTA-3’<br>Reverse: 5’- CAGAAGTCAGAGTGAAGGGATG-3’
Mouse *Nox4*	Forward: 5’- CCAGAATGAGGATCCCAGAAAG-3’<br>Reverse: 5’- GGTAGAAGCTGTAACCATGAGG-3’

Supplementary Table 2. List of genotyping primers

Gene	Primer
Mouse *Ldlr* $^{-/-}$	Forward: 5’-GCT CCA TAG GCT ATC TGC TC-3’<br>Reverse: 5’-GTG TCG TAG GAC AAG TTA GG-3’
Mouse *ApoE* $^{-/-}$	P1 : 5’-GCCTAGCCGAGGGAGAGCCG-3’<br>P2 : 5’-TGTGACTTGGGAGCTCTGCAGC-3’<br>P3 : 5’-GCCGCCCCGACTGCATCT-3’

Supplementary Table 3. List of commercial antibodies

Antibodies	Supplier	Identifier	Application
CD68	Abcam	ab125212	IHC 1:100
Mac2	Cedarlane	CL8942AP	IHC 1:200
α-SMA	Sigma-Aldrich	A2547	IHC 1:100
SM-MHC	OriGene	TA323338	IHC 1:100
SM22α	Abcam	Ab14106	IHC 1:200
Ki67	Abcam	Ab15580	ICC 1:100
KLF4	Abcam	Ab106629	IHC 1:100<br>WB 1:1000
CD11b(M1/70), PE	eBioscience	12-0112	FC
CD45(30-F11), Pacific Blue	eBioscience	MCD4528	FC
CD80(B7-1), PerCP Cyanine5.5	BioLegend	104721	FC
F4/80 (BM-8), APC	eBioscience	17-4801-82	FC
Human IgG Fc (HP6017)	BioLegend	409303	FC

WB, Western Blot; IHC, Immunohistochemistry; ICC, Immunocytochemistry; FC, Flow Cytometry

Supplementary Table 4. Baseline Clinical Characteristics

Factor	Low ANGPTL4 (n=26)	High ANGPTL4 (n=26)	P value
Age (years)	69.4±12.2	70.0±11.4	0.852
Men, n (%)	16 (61.5)	22 (84.6)	0.061
Systolic BP (mmHg)	123.1±40.8	119.6±19.7	0.280
Heart rate (/min)	85.9±20.6	81.7±20.9	0.466
Current or ex-smoker, n (%)	15 (57.7)	19 (73.1)	0.244
Hypertension, n (%)	19 (73.1)	11 (42.3)	**0.025**
Diabetes mellitus, n (%)	16 (61.5)	10 (38.5)	0.096
Dyslipidemia, n (%)	8 (30.8)	4 (15.4)	0.188
BMI (kg/m^2)	23.8±4.1	24.0±3.3	0.839
Obesity, n (%)	9 (34.6)	10 (38.5)	0.773
Familial history of CAD, n (%)	1 (3.8)	0 (0)	1.000
Previous MI, n (%)	3 (11.5)	5 (19.2)	0.703
Previous HF, n (%)	2 (7.7)	1 (3.8%)	1.000
ANGPTL4 levels (ng/mL)	1.53±0.72	4.64±2.69	**<0.001**
Serum creatinine (mg/dL)	1.5±1.3	1.3±1.0	0.545
Peak troponin-I (mg/dL)	60.1±79.0	68.6±62.0	0.253
Peak CK-MB (mg/dL)	57.1±91.2	93.0±100.1	0.210
Total cholesterol (mg/dL)	159.7±40.6	165.6±44.5	0.503
Triglyceride (mg/dL)	111.2±58.0	117.8±85.3	1.000
HDL-cholesterol (mg/dL)	42.9±10.3	41.9±12.6	0.515
LDL-cholesterol (mg/dL)	100.7±30.5	103.3±34.8	0.813
Serum glucose (mg/dL)	220.9±137.7	140.2±44.1	**0.012**
N-terminal pro BNP (pg/mL)	3,692.0±4825.3	7,169.3±11960.2	0.627
High-sensitivity CRP (mg/L)	3.6±5.9	2.3±4.0	0.493
Left ventricular EF (%)	45.8±10.9	48.5±10.7	0.374
Medications at discharge, n (%)			
Aspirin	26 (100)	26 (100)	1.000
Clopidogrel	18 (69.2)	11 (42.3)	0.051
Prasugrel or Ticagrelor	10 (38.5)	18 (69.2)	**0.026**
Beta-blocker	21 (80.8)	24 (92.3)	0.419
ACEi or ARB	24 (92.3)	24 (92.3)	1.000
Aldosterone antagonist	7 (26.9)	12 (46.2)	0.150
Statin	24 (92.3)	26 (100)	0.490

Values are presented as mean±SD, median [interquartile range] or number (percentage). ACEi, angiotensin-converting enzyme inhibitor; ARB, angiotensin-II receptor blocker; BNP, brain-type natriuretic peptide; BP, blood pressure; CAD, coronary artery disease; CK-MB, creatine kinase-myocardial band isoenzyme; CRP, C-reactive protein; EF, ejection fraction; HDL, high-density lipoprotein; LDL, low-density lipoprotein.

Supplementary Table 5. Coronary Angiographic and Procedural Characteristics

Factor	Low ANGPTL4 (n=21)	High ANGPTL4 (n=21)	P value
Culprit LAD, n (%)	12 (46.2)	17 (65.4%)	0.163
MVD, n (%)	13 (50.0)	13 (50.0)	1.000
ACC/AHA B2/C lesion, n (%)	26 (100)	25 (96.2)	1.000
Pre-PCI TIMI flow grade 0, n (%)	14 (58.3)	10 (38.5)	0.266
Symptom-to-balloon time (min)	2482.7±4130.7	1752.7±2865.6	0.260
Post-PCI TIMI flow grade 3, n (%)	26 (100)	26 (100)	1.000

Values are presented as mean±SD or number (percentage). ACC, American College of Cardiology; AHA, American Heart Association; LAD, left anterior descending; MVD, multivessel disease; PCI, percutaneous coronary intervention; TIMI, thrombolysis in myocardial infarction.

Supplementary Table 6. Factors Associated with Recurred Heart Failure

Factor	Crude Hazard Ratio on Univariate Analysis (95% CI)	P value	Adjusted Hazard Ratio on Multivariate Analysis (95% CI)	P value
Age (per year increase)	1.078 (1.026-1.133)	0.003	1.098 (1.023-1.179)	**0.010**
Male	1.730 (0.603-4.962)	0.308		0.326
Killip class 3	2.762 (1.012-7.537)	0.047	2.363 (0.254-22.007)	0.450
Hypertension	0.797 (0.488-1.302)	0.365		
Diabetes mellitus	0.936 (0.580-1.511)	0.788		
Dyslipidemia	0.848 (0.479-1.501)	0.571		
Obesity	2.250 (0.730-6.939)	0.158		
Smokers	0.775 (0.286-2.101)	0.616		
Family history	4.557 (0-190080.174)	0.780		
Previous HF	4.707 (0.020-1115.518)	0.579		
Previous MI	0.832 (0.444-1.559)	0.566		
Systolic blood pressure	1.003 (0.988-1.020)	0.670		
Heart rate	1.006 (0.985-1.026)	0.599		
Pain to balloon time	1.000 (1.000-1.000)	0.115	1.001 (1.000-1.001)	0.201
MVD	1.031 (0.396-2.685)	0.951		
Culprit LAD	0.872 (0.320-2.374)	0.788		
Creatinine	1.334 (1.202-1.745)	0.035	1.277 (0.854-1.908)	0.234
ANGPTL4 levels	0.465 (0.296-0.729)	0.001	0.338 (0.166-0.686)	**0.003**
Peak CK-MB	0.996 (0.990-1.002)	0.231		
Peak troponin-I	0.995 (0.986-1.003)	0.186	0.998 (0.981-1.014)	0.763
hsCRP	1.131 (1.024-1.249)	0.016	1.096 (0.973-1.234)	0.131
Initial LVEF	0.990 (0.942-1.040)	0.686		
Beta-blocker at discharge	0.549 (0.200-1.511)	0.246		
ACEi or ARB at discharge	0.042 (0.000-45.106)	0.373		
Aldosterone antagonist at discharge	1.338 (0.793-2.256)	0.275		
Use of statin at discharge	1.082 (0.393-2.977)	0.879		

ACEi, angiotensin-converting enzyme inhibitor; ANGPTL4, angiopoietin like 4; ARB, angiotensin-II receptor blocker; CAD, coronary artery disease; CI, confidence interval; CK-MB, creatine kinase-myocardial band isoenzyme; HF, heart failure; hsCRP, high-sensitivity C-reactive protein, LAD, left anterior descending; LVEF, left ventricular ejection fraction; MI, myocardial infarction; MVD, multivessel disease.

Full unedited gel for Figure 6C- KLF4 & β-actin

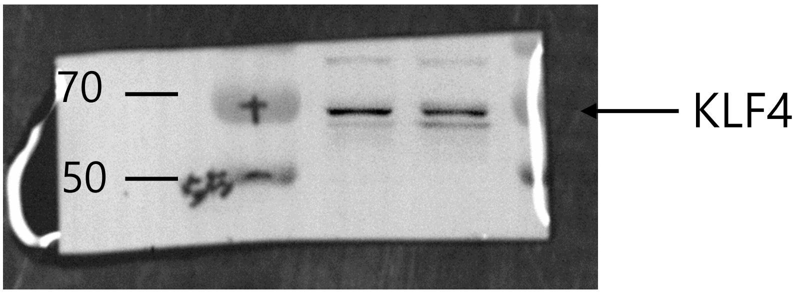

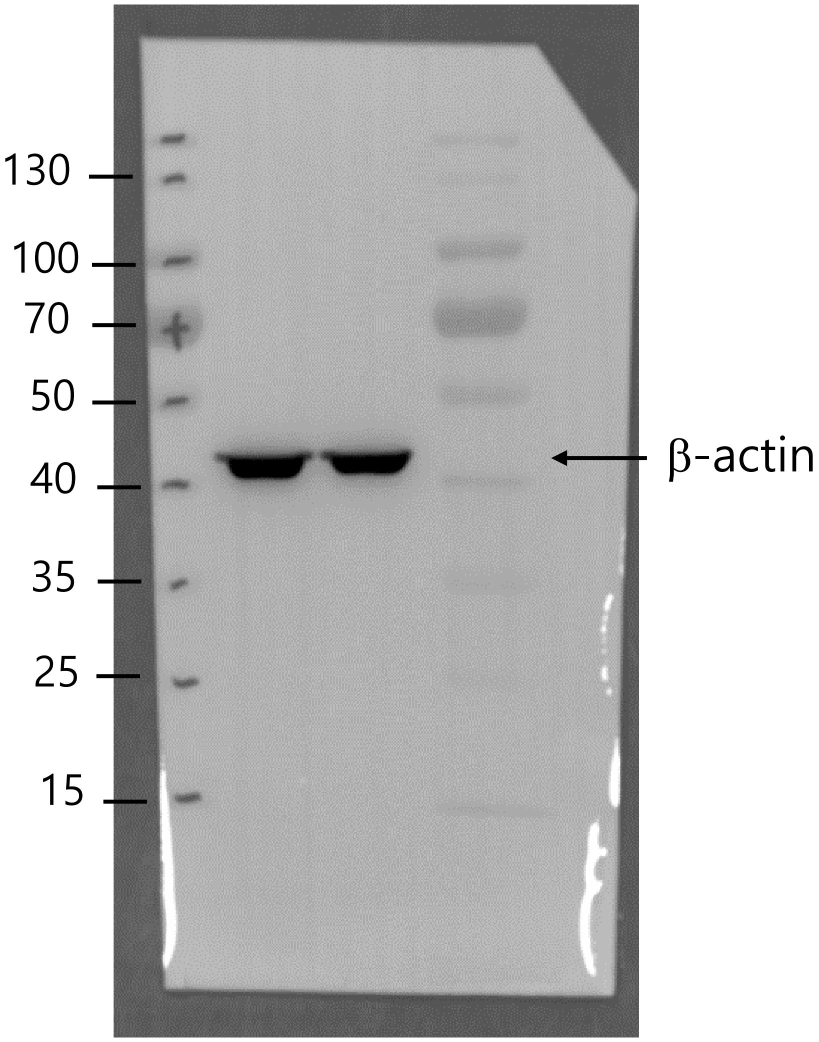

Full unedited gel for Figure 6D- KLF4 & GAPDH

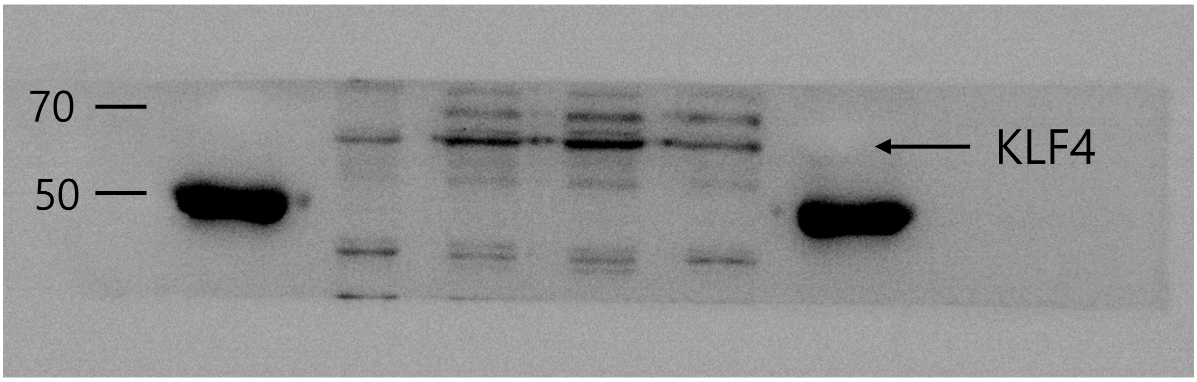

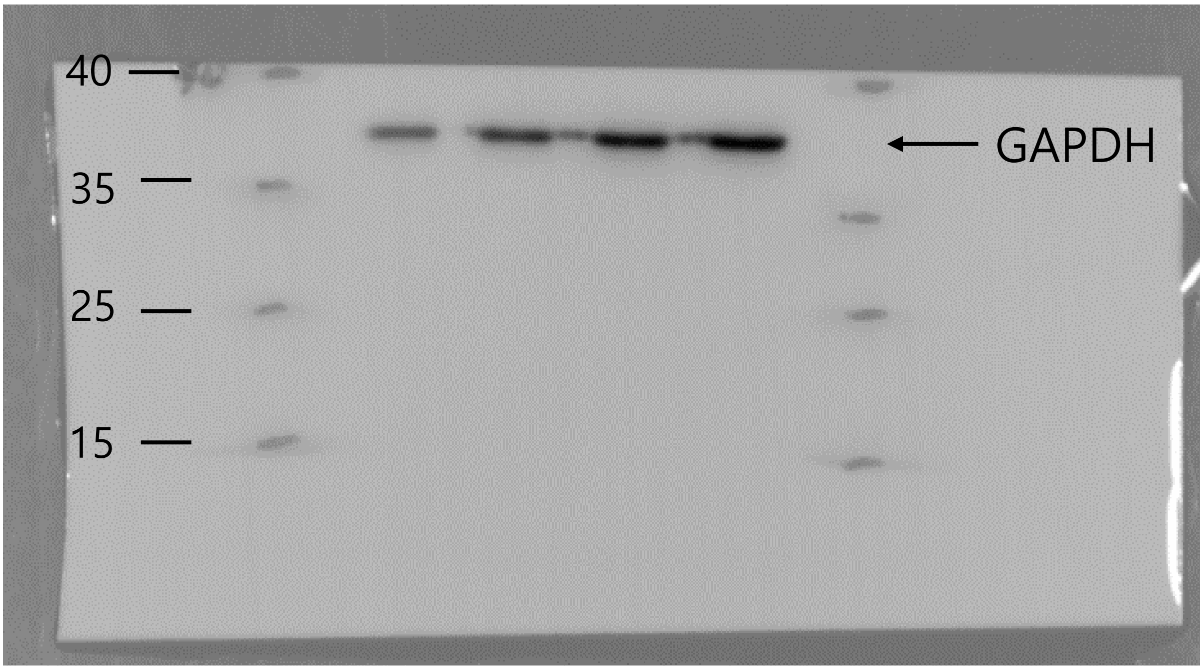

Supplement: Supplementary file 1 — Supplementary Information [file 12276_2023_937_MOESM1_ESM.pdf]
